# TEAD4 antagonizes cellular senescence by remodeling chromatin accessibility at enhancer regions

**DOI:** 10.1007/s00018-023-04980-9

**Published:** 2023-10-19

**Authors:** Donghui Zhang, Yanmei Zhu, Yanmin Ju, Hongyong Zhang, Xiaopeng Zou, Shangrong She, Danping Zhu, Yiting Guan

**Affiliations:** 1 Zhanjiang Institute of Clinical Medicine, Central People’s Hospital of Zhanjiang, Guangdong Medical University Zhanjiang Central Hospital, Zhanjiang, 524045 People’s Republic of China; 2 College of Pharmacy, China Pharmaceutical University, Nanjing, 211198 People’s Republic of China

**Keywords:** Chromatin accessibility, Enhancer, TEAD4, SASP, Senescence

## Abstract

Dramatic alterations in epigenetic landscapes are known to impact genome accessibility and transcription. Extensive evidence demonstrates that senescent cells undergo significant changes in chromatin structure; however, the mechanisms underlying the crosstalk between epigenetic parameters and gene expression profiles have not been fully elucidated. In the present study, we delineate the genome-wide redistribution of accessible chromatin regions that lead to broad transcriptome effects during senescence. We report that distinct senescence-activated accessibility regions (SAAs) are always distributed in H3K27ac-occupied enhancer regions, where they are responsible for elevated flanking senescence-associated secretory phenotype (SASP) expression and aberrant cellular signaling relevant to SASP secretion. Mechanistically, a single transcription factor, TEAD4, moves away from H3K27ac-labled SAAs to allow for prominent chromatin accessibility reconstruction during senescence. The enhanced SAAs signal driven by TEAD4 suppression subsequently induces a robust increase in the expression of adjacent SASP genes and the secretion of downstream factors, which contribute to the progression of senescence. Our findings illustrate a dynamic landscape of chromatin accessibility following senescence entry, and further reveal an insightful function for TEAD4 in regulating the broad chromatin state that modulates the overall transcriptional program of SASP genes.

**Electronic supplementary material:**

The online version of this article (10.1007/s00018-023-04980-9) contains supplementary material, which is available to authorized users.

## Introduction

Cells enter a senescent state in response to stress, which causes permanent cell cycle arrest and complex morphological, metabolic, and transcriptional alterations [1]. During senescence, cells display enhanced activity of lysosomal senescence-associated β-galactosidase (SA-β-gal), in addition to elevated expression levels of the tumor suppressor protein P16 [2, 3]. To date, the involvement of numerous tumor suppressor pathways, including p53/p21^CIP1^ and p16^INK4a^/RB, has been demonstrated in the senescence-related cessation of proliferation; however, the upstream portion of the regulatory network remains largely unknown [4–6].

Notably, the senescence-associated secretory phenotype (SASP) has become the most fundamental feature of cellular senescence, representing a substantial supply of secretory signals to the extra- and intracellular microenvironments for the initiation of inflammation and tissue-remodeling [7, 8]. Recent research has shown that the SASP helps to attract immune cells that aid in the removal of senescent cells from the tumor microenvironment, increasing the healthy lifespan of mice [9]. Nevertheless, the SASP can also play an immunosuppressive role by producing preneoplastic hepatocytes to inhibit immune cells or by reducing the levels of proinflammatory cytokines to decrease systemic inflammation [10, 11]. Considering the pleiotropic effects that the SASP exerts, clarifying the mechanisms that control the SASP is imperative to increase the targets available for the treatment of senescence-associated diseases.

Indeed, there is an entropy change during senescence that is based on the gene regulatory interplay between the cellular machinery and information stored in the genome and epigenome; nevertheless, passive or spontaneous changes in epigenetic modifications have been recognized as the most convincing putative contributor to the senescence process [12, 13]. Transgenerational inheritance mediated by epigenetic mechanisms has revealed that epigenetic drifts occur during senescence, and most crucially, that environmental and epigenetic variables can directly regulate senescence kinetics [14]. For example, studies in simple organisms have indicated that a loss of epigenomic information may be an underlying cause of aging [15, 16]. The manipulation of DNA methylation, histone post-translational modification, chromatin remodeling, and non-coding RNA expression has been shown to be vital for the function of chromatin to regulate the accessibility of DNA to bind transcriptional complexes [17–20].

As a significant component of epigenetic control, chromatin accessibility mediated by nucleosome placement participates in the regulation of gene networks. The cooperation of chromatin accessibility with regulatory elements, such as H3K27ac-decorated enhancers, fundamentally alters the chromatin state, which in turn affects the downstream transcriptional program [21]. Remarkably, as the most significant genetic feature impacting biological behavior at a specific locus, accessible chromatin allows transcription factors to target closed chromatin by opening up these regions. Application of the ground-breaking epigenetic assay for transposase-accessible chromatin with sequencing (ATAC-seq) technology has enhanced our knowledge regarding the mechanisms governing epigenetic alteration and the control of gene expression during fundamental biological processes [22–24]. Previous reports have demonstrated that dynamic alterations in the chromatin landscape are correlated with distinct SASP transcriptional profiles. The enhancer landscapes are redistributed through the recruitment of BRD4, a member of the bromodomain and extra-terminal (BET) family, to chromatin regions with active transcription, thus activating flanking SASP genes [25]. In another example, the suppression of high mobility group box 2 (HMGB2) can disrupt the chromatin landscape of SASP factors by allowing the spread of repressive heterochromatin into gene loci [26]; therefore, the control of gene expression profiles by the modulation of chromatin accessibility may be able to predict cell fate and stimulus response [27]. Considering that the cellular senescence regulatory network is modulated by hierarchical epigenetic parameters, dissecting the dynamic interplay between the chromatin landscape and gene expression programs will aid the development of effective treatments for senescence-associated diseases.

Here, we demonstrate that widespread chromatin accessibility remodeling occurred during replicative senescence, which cooperated with the redistribution of senescence-driven enhancer repertoires. A significant increase in accessible sites, defined as senescence-activated accessibility regions (SAAs), were shown to facilitate the expression of neighboring SASP genes. Moreover, the transcription factor TEAD4 was found to be enriched at SAAs regions and eviction of TEAD4 acted as a prerequisite for downstream chromatin accessibility establishment, while TEAD4 suppression accelerated the expression and secretion of SASP as well as the progression of senescence. Taken together, our findings reveal the role of TEAD4-driven chromatin accessibility remodeling in the gene regulatory network, which provides therapeutic opportunities for treating senescence-associated diseases.

## Materials and methods

### Human material and cell culture

Primary human umbilical cord mesenchymal stem cells (UC-MSCs) were obtained from fetuses during natural labor at Central People's Hospital of Zhanjiang. The experiment was approved by the Institutional Ethics Committee of Central People's Hospital of Zhanjiang (IIT-2022–057-01). Briefly, the fresh umbilical cord was cut into pieces and digested with 1 g/L collagenase II for 1 h. The disassociated cells were cultured at 37 °C under 5% CO_2_ in DMEM supplemented with 20% fetal bovine serum (FBS, Gibco, 12,800), 100 U/mL streptomycin, and 100 U/mL penicillin. Cell clumps were carefully removed after 16 days, and the remaining cells were considered population doubling 0 (PD0). A total of 1 × 106 cells were transferred to a 100-mm dish and cultured until confluence to generate PD1. The PD was calculated using PD = log2 (Ne/N0), where N0 indicates seed number and Ne indicates count number. Cumulative PDs were calculated as the total PD numbers from all prior passages.

HEK 293 T cells (ATCC, CRL-3216) were cultured in DMEM supplemented with 10% FBS (Gibco, 12,800), 100 U/mL streptomycin, and 100 U/mL penicillin under 5% CO_2_ at 37 °C.

### Cellular senescence assays

For immunofluorescence, cells were cultured on flow slides in dishes. After reaching a confluence of 80%, cells were subjected twice to washing in ice-cold PBS and subsequently fixed for 10 min in ice-cold 4% paraformaldehyde. Slides were then washed in PBS, blocked for 1 h at room temperature in 1% BSA, and incubated at 4 °C overnight with an anti-Ki-67 primary antibody (Abcam, ab15580; 1:200) in blocking buffer. The next day, cells were washed and then incubated at room temperature for 2 h with secondary antibody. DAPI Fluoromount-G was used to mount the slides, which were subsequently imaged using a fluorescence microscope. ImageJ was used to quantitate the nuclear fluorescence intensities.

A commercial senescent cell histochemical staining kit (Sigma-Aldrich, CAS0030) was used for the SA-β-gal assay. Briefly, cells were seeded at a density of 2 × 10^5^ cells/well onto 6-well plates, allowed to adhere, and treated under the indicated conditions. Cells were then fixed for 5 min and incubated overnight at 37 °C in the dark with β-Gal staining solution. Light microscopy was used to count the blue stained senescent cells and ImageJ was employed for quantitation. A 75% glycerin/PBS solution was added to the plates for long-term storage.

### Expression analysis

For immunoblotting, cell lysates were obtained by incubation in RIPA buffer (Sigma–Aldrich, R0278). Bradford reagent was used to determine protein concentration for normalization of loading. Proteins were resolved by SDS-PAGE, transferred to nitrocellulose membrane, and visualized by immunoblotting.

Total RNA was isolated from cells using TRIzol reagent (Invitrogen, 10,296,028). For RT-qPCR analysis, an RT reagent kit (TaKaRa, RR037Q) was used to reverse-transcribe 500 ng total RNA into cDNA. The reaction was carried out in a total volume of 20 μL containing 2 μL cDNA and 10 μL 2 × Green qPCR SuperMix (TransGene, AQ601) using a LightCycler 450. *GAPDH* mRNA was used as the internal control for normalization. Primer sequences are provided in Supplementary Information.

For RNA-seq analysis, the library was sequenced on the Novaseq PE150 platform. Clean reads were obtained by removing adapter sequences and low-quality data. The HISAT2 software (version 2.1.0.) was used to compare the clean reads with the hg38 reference genome, and reads within each gene were counted and normalized according to DESeq2, |log2FC|> 1 and *p* < 0.05.

For cytokine detection, a human XL cytokine array kit (R&D Systems, ARY022B) was used to analyze the supernatants from serially passaged cells.

### Antibodies

All antibody information is provided in Supplementary Information.

### ATAC-seq and data processing

ATAC-seq was carried out as previously described with minor modifications [24]. Briefly, cells were trypsinized to create a single-cell suspension. A total of 50,000 nuclei were isolated, and Tn5 transposome and tagmentation procedures were performed at 37 °C for 30 min (Vazyme, TD501). Samples were purified using 2 × AMPure beads (Beckman Coulter, A63882) and subjected to 14 cycles of PCR for library amplification. The conditions were as follows: 72 °C for 3 min; 98 °C for 30 s; and thermocycling at 98 °C for 15 s, 60 °C for 30 s, and 72 °C for 30 s; followed by 72 °C for 5 min. Product fragments were screened and then purified using 0.65 × and 0.8 × AMPure beads. Finally, libraries were sequenced on the Novaseq PE150 platform to a depth of 4.0 × 10^7^ reads.

For ATAC-seq data processing, the raw reads were removed from the adaptor sequences using Trimmomatic, and then the BWA-MEM software was employed to align the reads to the hg38 human reference genome. Significant ATAC-seq peaks from a bam file of uniquely mapped reads were called by ChIPseq (MACS2 v2.1.2). The cutoff value for peak calling was *q* = 0.05. To identify SAAs and SIAs, a consensus set of unique peaks was created by merging the ATAC-seq peaks in each sample. Bedtools v2.25.0 was then used to count the number of peaks, and DESeq2 (version 1.16.0) was employed with the thresholds |log2FC|> 2 and *p* < 0.05 to identify differential peaks. In addition, the HOMER and MEME algorithms were used to search for motif enrichment in SAAs and SIAs regions. Bam Coverage in deepTools (version 3.3.0) was used to create genome-wide normalized signal coverage tracks, and Integrative Genomics Viewer (version 2.5.0) was used for visualization.

### CUT&Tag and data processing

The Hyperactive® Universal CUT&Tag Assay Kit for Illumina (Vazyme, TD903) was used for CUT&Tag analysis. In brief, nuclei were extracted from 10,000 fresh cells and incubated with ConA beads for 10 min at room temperature. The complexes were incubated overnight with primary antibodies against H3K27ac and TEAD4, and an IgG antibody served as the negative control. The next day, the complexes were incubated sequentially with a secondary antibody and a diluted pA-Tn5 adapter complex for 1 h at room temperature. After extensive washing, shearing was performed using 5 × TTBL, and DNA was eluted using proteinase K and DNA extraction beads. Finally, the sequencing libraries were established following 12 cycles of PCR amplification. The selected DNA fragments were sequenced on the Novaseq PE150 platform to a depth of 2.5 × 10^7^ reads. For data processing, FASTQC was used to measure the data quality distribution, and the bwa program was employed to align the clean reads to reference genome sequences. The MACS2 software was used with a *q*-value threshold of < 0.05 for calling peaks and screening. IGV was used to visualize genome-wide normalized signal coverage tracks. Peaks with greater than a twofold change and false discovery rate < 0.1 were considered decreased, while those with a log2 fold change not significantly different from zero were termed constant.

### ChIP-qPCR analysis

ChIP was carried out as described previously [28]. Briefly, crosslinking of the cells was performed for 10 min using 1% formaldehyde solution, after which glycine was used to stop the reaction. Chromatin was sheared into 300–500-bp fragments and precleared using 20 μL Protein A Sepharose beads and salmon sperm DNA (Sigma, GE17-5280) by rotation for 30 min at 4 °C. A 250-μg fraction of the beads/DNA mixture was incubated overnight with rotation at 4 °C with 3 μg primary antibody. The following day, 50 μL Protein A Sepharose bead slurry was used for pulldown. Following extensive washing, the complexes were eluted and subsequently de-crosslinked for 5 h at 65 °C. Finally, ChIP DNA was purified by phenol chloroform extraction for qPCR. The ChIP-qPCR primers are provided in Supplementary Information.

### RNAi and cDNA transfection

TEAD4 and YAP were knocked down using small-interfering RNAs (siRNAs) by transfection of UC-MSCs at a final concentration of 100 μM using Lipofectamine™ 3000 (Invitrogen, L3000015) according to the manufacturer’s instructions. siRNA sequences are provided in Supplementary Information.

For TEAD4 overexpression, TEAD4 cDNA was integrated into the pSIN-GFP vector, which was then used to transfect UC-MSCs by the Lipofectamine™ 3000 method for 48 h. Transfection efficiency was examined by measuring the density of GFP + cells after 24 h. The pSIN-GFP vector was a kind gift from Prof. Zhengfan Jiang from Peking University. The pcDNA3.1-YAP-Flag plasmid was purchased from Vigene Biosciences corporation (CH854403).

### Immunoprecipitation

In brief, cells were collected after washing three times in cold PBS and lysed with ELB buffer on ice for 30 min. The lysates were centrifuged at 20,000×*g* for 20 min at 4 °C, and the supernatants were mixed with the related antibodies at 4 °C for 2 h, and then incubated with Protein A/G Sepharose beads for a further 2 h. After extensive washing, the beads were boiled at 100 °C for 10 min in SDS loading buffer. Subsequently, immunoblotting was performed to examine the interactions between exogenous and endogenous proteins.

### Luciferase reporter assay

The luciferase backbone vector is modified PGL4.10 (Promega, E665A), into which the SAA fragments for *IL6* were cloned upstream of the luc2 reporter gene. HEK 293T cells were seeded on 24-well plates, cultured to 80% confluence, and then co-transfected with 200 ng constructed luciferase plasmid, 200 ng pSIN-TEAD4 plasmid, and 8 ng Renilla plasmid using Lipofectamine™ 3000 (Invitrogen, L3000015). At 48 h post-transfection, cells were harvested and assayed using the Dual Luciferase Reporter Assay System (Promega, E1910). The relative luciferase activity was normalized to that of Renilla luciferase activity. Primers for the cloning of SAA fragments are provided in Supplementary Information.

### Statistical analysis

Data are expressed as the mean ± SEM. Data were analyzed using a two-tailed unpaired Student’s *t*-test or one-way ANOVA followed by Dunnett’s multiple comparison test (GraphPad Prism software, version 5.01). *p* < 0.05 was considered statistically significant.

## Results

### Serially passaged cells display distinct chromatin accessibility profiles upon senescence entry

**Fig. 1:**
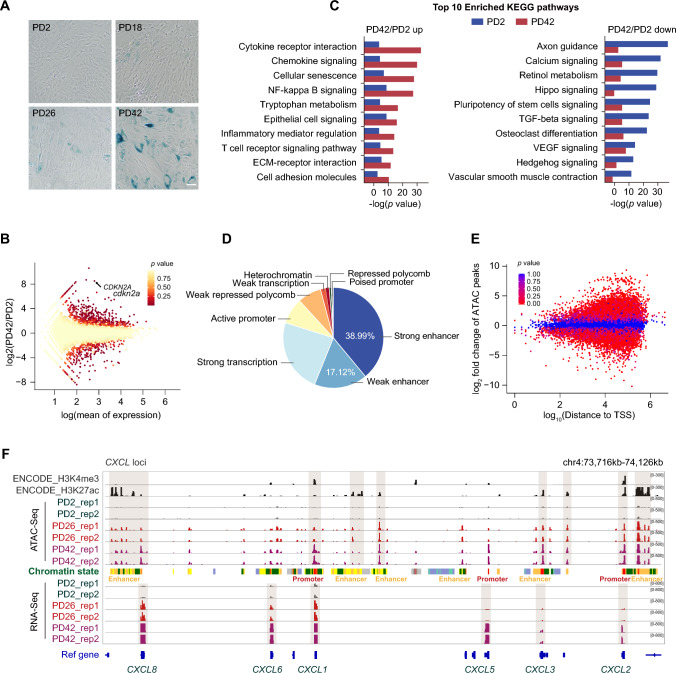
Remodeling of the open chromatin landscape and transcriptional network occur during UC-MSC senescence. **A** SA-β-gal staining of cells at different stages from proliferation to senescence. PD2, young cells; PD26, midterm cells; and PD42, senescent cells. Scale bar, 50 μm. **B** RNA-seq scattergram of UC-MSCs showing the gene expression profiles at PD42 versus PD2. **C** Top-ranked KEGG enrichment scores of up/downregulated genes from the transcriptome dataset at PD42 versus PD2. **D** Annotations of all open chromatin regions showing the chromatin states trained using the public UC-MSC data from the ENCODE project. **E** Scatter plot of ATAC-seq peaks for UC-MSCs showing the change in accessibility between PD42 and PD2. TSS, transcription start site. **F** Integrative Genomics Viewer (IGV) snapshot displaying the H3K4me3 and H3K27ac peaks of MSCs from ENCODE, and the ATAC-seq and RNA-seq peaks from young to senescent UC-MSCs at the *CXCL* loci. There were two independently performed biological replicates for each passage number (rep 1 and 2). Vertical gray boxes indicate enhancer and promoter ATAC-seq peaks. Chromatin states were obtained from ENCODE: yellow, weak enhancer; orange, strong enhancer; red, active promoter; green, transcribed region; and gray, heterochromatin

A cellular senescence model was established by serial passage of human umbilical cord-derived mesenchymal stem cells (UC-MSCs). In comparison with young cells, the long term-cultured UC-MSCs displayed a characteristic senescence phenotype, which included a reduced proliferation rate, decreased Ki-67 staining, increased senescence-associated β-galactosidase (SA-β-gal) staining, and increased P16 (encoded by *CDKN2A*) protein expression (Fig. 1A, Fig. S1A–D). The acquisition of SASP factors that alter the microenvironment of cell populations is one of the most prominent features of senescent cells [29]. Here, we examined the SASP secretome using commercially available cytokine arrays and found that many prominent cytokines of the SASP, such as IL-6, IL-8, CXCL-5, IGFBP2, IGFBP3, and MCP-3, were accumulated in the supernatant of senescent cells (Fig. S1E, F).

To determine the global gene expression profiles following senescence entry, two highly reproducible independent replicates from three population doublings (PDs) of UC-MSCs were subjected to RNA-seq: PD2 (young cells), PD26 (midterm cells), and PD42 (senescent cells) (Fig. S2A). Results of distinct hierarchical clustering analysis suggest that the senescent state significantly impacted the genome-wide transcriptome profile, and the expression level of the well-recognized senescence marker gene *CDKN2A* was significantly elevated in senescent cells [30] (Figs. 1B, S2B and C). Gene set enrichment analysis (GSEA) data further indicate that metabolic signaling, organogenesis, and other developmental processes were downregulated during senescence (Fig. S2D and E). In addition, the downregulated genes were involved in development-related pathways, while the upregulated genes were mainly enriched in the SASP-mediated immune response pathway, which supports the rewiring of a subset of SASP genes during senescence (Fig. 1C).

Emerging evidence has enabled us to begin to understand the organization of chromatin accessibility and the manner by which it defines regulatory elements within the genome to cooperatively control gene expression [27]. Given the unique alterations in SASP genes and the enrichment of senescence-associated pathways, we performed two reproducible and independent ATAC-seq analyses at different PD times to investigate the manner by which transcriptional alterations underlying senescence are associated with chromatin accessibility (Fig. S3A). Most of the fragments were consistent with typical inter-nucleosome open chromatin mapping, and ATAC-seq signals were enriched around transcription start sites (TSS), demonstrating that our data are both reliable and of high quality (Fig. S3B and C). Moreover, senescent cells displayed decreased chromatin accessibility in comparison with young cells, which is inversely associated with the previously reported increase in chromatin compaction [31] (Fig. S3D). The subsequent genomic distribution patterns show that the chromatin accessibility regions were always located in *cis*-regulatory element-enriched regions, with genome annotation further implying that a large fraction of these peaks overlapped with enhancer signals (Fig. 1D, Fig. S3E). Accordingly, ATAC-seq peaks were observed far from TSSs, where the proportion of enhancers is the largest (Fig. 1E). Therefore, we compared our data with the public Encyclopedia of DNA Elements (ENCODE) to track ATAC-seq peaks using epigenomic profiling. Indeed, these ATAC-seq signals tended to be distributed within enhancer regions decorated by H3K27ac. For instance, the expression levels of prominent SASP genes, *CXCLs*, *IL6*, and *IL1B*, were significantly increased during senescence, exhibiting the most prevalent ATAC-seq signals in active enhancer regions decorated by H3K27ac but not in promoter regions decorated by H3K4me3, suggesting that strong and active enhancer occupancy is a chromatin accessibility signal upon senescence entry (Fig. 1F, Fig. S4A and B).

### Senescence-associated differentially accessible regions display unique enrichment with enhancer regions

**Fig. 2:**
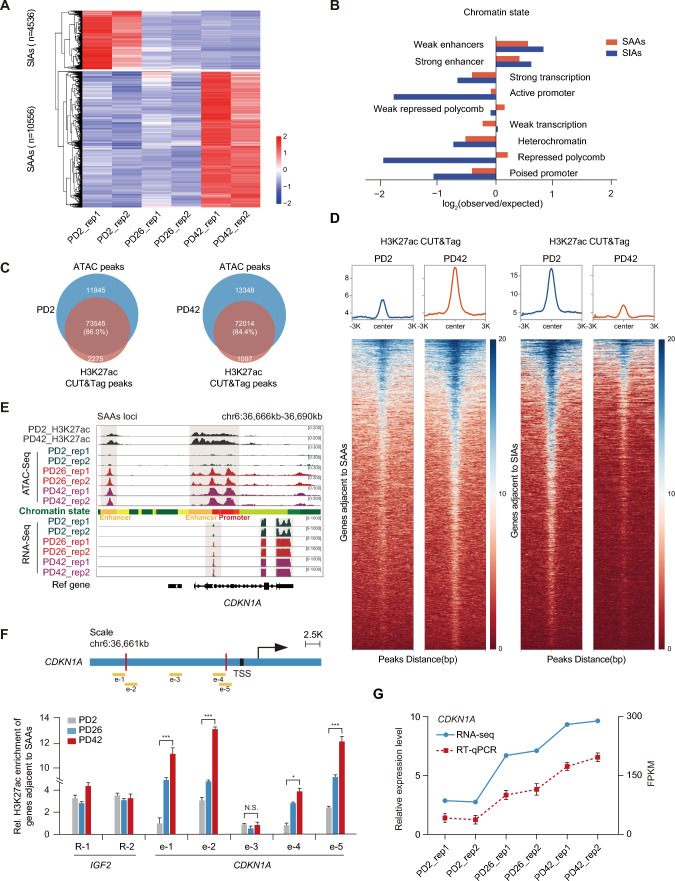
Senescence-activated accessibility regions (SAAs) and -inactivated accessibility regions (SIAs) are often distributed in enhancer-enriched regions. **A** Heatmap of hierarchically clustered ATAC-seq data in young to senescent UC-MSCs, displaying the remodeling of SAAs and SIAs. **B** The defined enrichment of chromatin states at SAAs and SIAs using ENCODE public data. **C** Venn diagram showing the overlap of ATAC-seq peaks with H3K27ac CUT&Tag peaks. **D** Heatmaps and enrichment plots showing normalized read densities of H3K27ac CUT&Tag peaks for genes adjacent to SAAs and SIAs at transcription start sites (TSSs) at PD2 and PD42. Tracks are centered at the peaks and extend ± 3 kb. **E** Snapshot displaying the H3K27ac, ATAC-seq, and RNA-seq profiles of young to senescent cells at representative SAA regions within the *CDKN1A* locus. Vertical gray boxes indicate ATAC-seq peaks in enhancer and promoter regions and the corresponding *CDKN1A* expression. **F** Location diagram of H3K27ac ChIP-qPCR primers within the *CDKN1A* locus (top). ChIP-qPCR was used to measure the relative H3K27ac levels for SAAs within the corresponding *CDKN1A* gene in young to senescent cells (bottom). H3K27ac enrichment in *IGF2* was used as the positive control, and IgG was used as the negative control. The enrichment was normalized to a 1:10 dilution of the input. Error bars indicate the mean ± S.E.M. of three independently performed experiments. One-way ANOVA followed by Dunnett’s multiple comparisons test was used for statistical analysis. N.S., not significant, *p < 0.05, ***p < 0.001. **G** Expression of *CDKN1A* in young to senescent cells. The dashed line indicates RT-qPCR, while the solid line indicates RNA-seq data. Error bars indicate the mean ± S.E.M. of three independently performed experiments

Although chromatin regions and gene expression in different stages of senescence share common characteristics, the senescence-associated differentially accessible regions (DARs) are crucial to the senescence process. In our research, > 10,000 DARs based on quantitative peak signals were rearranged during senescence, which included 10,556 senescence-activated accessibility regions (SAAs) and 4,536 senescence-inactivated accessibility regions (SIAs) (Figs. 2A, S5A). As expected, these two groups of DARs were primarily located in intergenic regions distributed approximately 2 kb from TSSs (Fig. S5B and C). In particular, assessment of the chromatin state of SAAs and SIAs across the genome uncovered that the expected enhancer elements were always included in the public epigenomic profiles, reinforcing the observation of enhancer element occupancy in the ATAC-seq peaks mentioned above (Fig. 2B).

Given that enhancers reside in dynamic DARs and have the potential to modulate the gene regulatory network, we explored the enhancer occupancy during senescence using H3K27ac-driven CUT&Tag technology. Overall, there was no significant difference in active enhancer signal across the genome between young and senescent cells (Fig. S5D and E); however, a large fraction (> 80%) of ATAC-seq peaks highly overlapped with H3K27ac-mediated enhancer peaks (Fig. 2C). Evaluation of the H3K27ac signal in genes adjacent to SAAs and SIAs revealed that SAAs flanking genes displayed increased H3K27ac signals in senescent cells, while SIAs flanking genes exhibited decreased H3K27ac levels after senescence entry, which is consistent with our findings regarding the global chromatin open state (Fig. 2D). As a vital senescence marker, *CDKN1A* was flanked by SAAs that showed substantial elevations in both chromatin accessibility and expression level during senescence (Fig. 2E). However, *EMP1*, a key regulator of angiogenesis that was found to be adjacent to SIAs, displayed decreased enhancer signals and gene expression during senescence [32] (Fig. S5F). Verification of the H3K27ac peaks and gene expression near *CDKN1A* and *EMP1* also highlighted that a high proportion of ATAC-seq peaks were occupied by H3K27ac (Figs. 2F, G, S5G,H). In essence, these data reveal that the chromatin accessibility profile is reorganized during senescence, while the distinctive differentially accessible regions are always distributed in H3K27ac-decorated enhancer regions.

### SASP genes are expressed adjacent to senescence-activated accessible regions (SAAs)

**Fig. 3:**
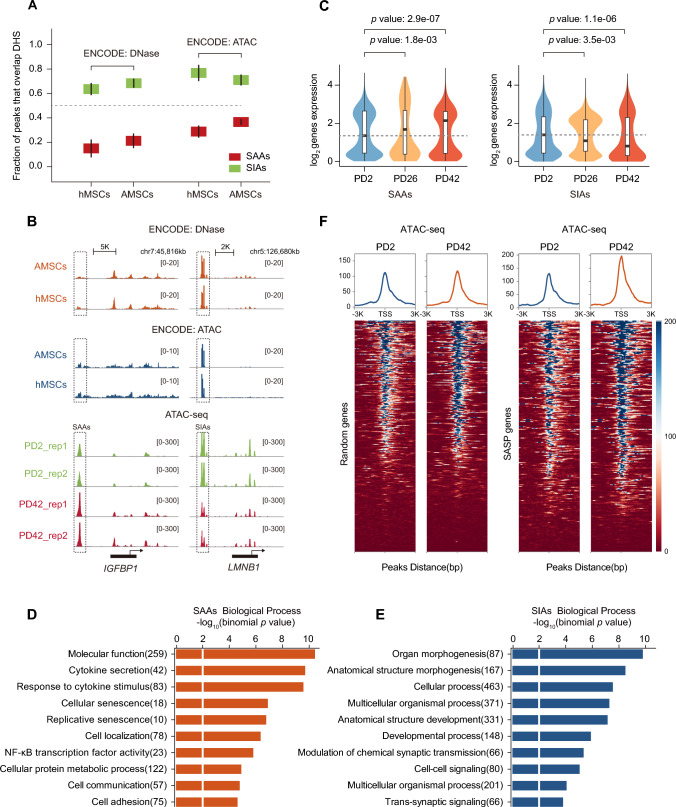
SAAs affect adjacent SASP gene expression profiles and SASP-related signaling pathways. **A** Overlap of SAAs and SIAs with DNase-seq and ATAC-seq peaks from public ENCODE AMSC and hMSC data. Mean overlap of peak calls with DNase I hypersensitive sites (DHSs) are shown. Bars represent 95% confidence intervals. AMSCs: adipose mesenchymal stem cells. hMSCs: human mesenchymal stem cells. **B** Integrative Genomics Viewer (IGV) snapshot showing the DNase-seq and ATAC-seq data from public ENCODE AMSCs and hMSCs, in addition to ATAC-seq signals at PD2 and PD42 at the representative SASP *IGFBP1* and nuclear architecture *LMNB1* loci. **C** Violin plots displaying the distributions of expression changes in SAAs- and SIAs-adjacent genes. **D** and **E** Gene Ontology enrichments of **D** SAAs and **E** SIAs using Genomic Regions Enrichment of Annotations Tool (GREAT) analysis. Bar length represents the enriched *p* value for biological processes. **F** Heatmaps and enrichment plots showing normalized read densities of ATAC-seq peaks for randomly selected genes (*n* = 350, left) and SASP genes (right) at the transcription start sites (TSSs) at PD2 and PD42. Tracks are centered at the TSS and extend ± 3 kb

To determine whether these two groups of DARs defined in senescence are common to other cell types, we compared our chromatin accessibility data to ENCODE DNase-seq and ATAC-seq data for other types of mesenchymal stem cells. We found that SIAs had the greatest overlap (> 50%) with hMSCs and AMSCs; however, SAAs were not significantly enriched in any specific cell type, suggesting a unique pattern of SAAs in senescence (Figs. 3A, S6A, B). For example, *LMNB1* involved in maintaining nuclear architecture was found adjacent to SIAs and displayed a strong but similar pattern of open regions across the cell types; whereas *IGFBP1*, a member of the SASP family that flanks SAAs, exhibited increased chromatin accessibility during senescence, in addition to quiescent signals in hMSCs and AMSCs (Fig. 3B). It appears that lineage-specific SAAs are involved in a distinctive remodeling of opening chromatin that facilitates the activation of flanking genes. Based on this hypothesis, we sought to elucidate whether enhancer-anchored SAAs can modulate the expression of nearby genes during senescence. Indeed, the genes adjacent to SAAs were found to always be upregulated during the process of senescence, while those near SIAs tended to be downregulated, indicating that both upregulation and downregulation of gene expression are invariably correlated with chromatin opening at active enhancers (Fig. 3C).

Subsequently, we performed gene ontology (GO) enrichment analysis to explore the biological functions of DAR-adjacent genes. Notably, the pathways relevant to SAAs were involved in the regulation of growth factors, NF-κB signaling, and cytokine secretion, which are vital for SASP control (Fig. 3D). In agreement with the GO enrichment analysis, many genes adjacent to SAEs were upregulated during senescence, including the well-described SASP genes (Fig. S7A). Meanwhile, the SIAs-enriched pathways were associated with developmental dysregulation and growth capacity exhaustion during senescence (Fig. 3E). Since a subset of SASP genes were increased during senescence among genome-wide transcriptome profiling, we quantitated the ATAC-seq peak intensities of recorded SASP genes. In comparison with the randomly selected genes, SAAs displayed significant SASP gene enrichment nearby, suggesting a potential regulatory role for SAAs in harboring adjacent SASP genes (Fig. 3F). In addition, a luciferase reporter assay was applied by expressing the individual constituent SAA fragments of *IL6* with a view to assessing whether SAAs modulate the expression of adjacent SASP genes. The constructed luciferase plasmids exhibited higher activity in comparison with the empty vector control (Fig. S7B). Meanwhile, the E4 and E5 constituent SAA regions produced the highest signal intensity followed by the E1 and E2 enhancers, while E3 displayed no significant change in signal intensity. Taken together, our results suggest that the SAAs-harboring SASP genes are accompanied by multi-SASP-controlled impaired signaling. Moreover, the newly activated SAAs are tightly coupled to a robust increase in the expression of adjacent SASP genes, which may serve as a prerequisite or influencing factor for aberrantly elevated SASP expression that contributes to cellular senescence.

### Eviction of TEAD4 from SAAs is responsible for the high expression of SASP genes during senescence

**Fig. 4:**
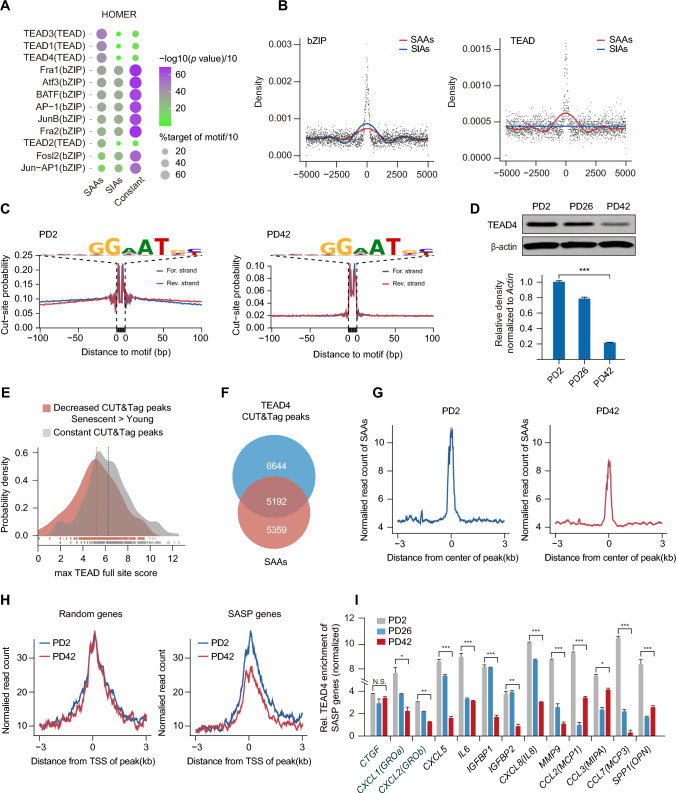
Movement of TEAD4 from SAAs leads to increased SASP gene expression. **A** Top-ranked enriched motifs among SAAs and SIAs were determined using Homer2 algorithms. The circle size represents the percentage of regions with motifs, and the color represents the *p*-value (Shrink to 10 ×). **B** Position-weight matrix displaying the observed accessibility densities in bZIP and TEAD motif families. **C** ATAC-seq footprint at the TEAD motif site at PD2 and PD42. Normalization was performed to ensure that each motif possessed the same mean number of insertions 200–500 bp away. **D** Immunoblotting analysis and quantitation of total TEAD4 expression during senescence. β-actin served as the loading control. ***p < 0.001. **E** Distribution of motif scores for sites within TEAD4 CUT&Tag peaks; those with either lower signals in senescent cells than in young cells (decreased) or those that were not significantly changed (constant). The maximum-scoring TEAD full site within each CUT&Tag peak was used. The constant peaks have a higher mean motif score than the differential peaks (Mann–Whitney U test, *p* < 1 × 10^−28^). **F** Venn diagram showing the overlap of TEAD4 CUT&Tag peaks and SAAs signals defined within ATAC-seq peaks. **G** Enrichment of TEAD4 CUT&Tag peaks in SAAs at both PD2 and PD42. Tracks are centered at the peaks and extend ± 3 kb. **H** Enrichment plots showing normalized read densities of TEAD4 CUT&Tag peaks at transcription start sites (TSSs) for randomly selected genes (n = 350, left) and SASP genes (right) at PD2 and PD42. Tracks are centered at the TSS and extend ± 3 kb. **I** TEAD4 ChIP-qPCR was used to measure the relative levels of representative SASP genes flanking SAAs during senescence. TEAD4 enrichment for *CTGF* was used as the positive control. Enrichment of TEAD4 was normalized to a 1:10 dilution of the input. One-way ANOVA followed by Dunnett’s multiple comparisons test was used for statistical analysis. *p < 0.05, **p < 0.01, ***p < 0.001

Chromatin accessibility allows access of transcription factors to specific sites that participate in cell fate decisions. To evaluate the potential drivers that modulate SAAs and alter the expression levels of downstream SASP genes, we examined transcription factor motif enrichment within SAAs and found significant enrichment of a number of distinct motifs in SAAs using the HOMER and MEME algorithms. Indeed, the top two most highly-ranked motifs shared significant homology with consensus binding sites for the basic leucine zipper domain (bZIP) and TEA domain transcription factor (TEAD) (Figs. 4A, S8A). Interestingly, the bZIP family motif was enriched in both SAAs and SIAs, while the TEAD motif was only strongly enriched in SAAs (Fig. 4B). Moreover, we observed a distinct ATAC-seq fragment pattern surrounding the TEAD motif in young cells relative to the open chromatin state in senescent cells, which was different from the constant bZIP signal during senescence seen by footprint analysis (Figs. 4C, S8B). These results indicate that a shift in TEAD occupancy resulted in the global open chromatin state at SAAs, suggesting a long-lived TEAD-DNA interaction prior to senescence.

Since the TEAD motif is enriched in SAAs, we searched for transcription factors that showed trends in expression changes with a view to identifying those that may be involved in regulating SAAs. Remarkably, only TEAD4 exhibited continuous downregulation at both the mRNA and protein levels during senescence (Figs. 4D, S8C). To further determine whether TEAD4 is involved in the remodeling of chromatin landscapes, we then assessed genome-wide TEAD4 occupancy by conducting TEAD4 CUT&Tag in young and senescent cells. Overall, TEAD4 displayed significantly decreased signals during senescence, which is in accordance with the decreased expression of TEAD4 observed upon senescence entry (Fig. S8D). Further, we quantitated and compared the motif scores for the CUT&Tag peaks between young and senescent cells. On average, the peaks that were decreased in senescent cells had lower motif scores than constant peaks during senescence, indicating that high TEAD4 expression levels in young cells enable binding to lower affinity sites, which is in accordance with the decreased expression of TEAD4 during senescence (Fig. 4E). Statistics for the profiling of TEAD4 with respect to SAAs also unveiled that more than a third of TEAD4 binding sites comprised SAAs, while the same proportion of more than a third of decreased TEAD4 binding sites in senescent cells relative to young cells were targeted by SAAs (Figs. 4F, S8E). Therefore, we assessed TEAD4 enrichment in SAAs and found reduced densities of TEAD4 in SAAs in senescent cells, which emphasizes the function of TEAD4 in the remodeling of SAAs (Fig. 4G). Since we discovered that SAAs are responsible for harboring downstream SASP genes, we speculated whether the departure of TEAD from SAAs facilitates the regulation of SASP genes. Indeed, SASP genes exhibited reduced TEAD4 enrichment flanking their TSSs, while a randomly selected set of genes displayed unchanged TEAD4 peaks during senescence (Fig. 4H). Subsequently, we performed ChIP-qPCR of TEAD4 to examine the signal around SASP genes that had been identified to be adjacent to SAAs. We found that the signal strength of TEAD4 around these SASP genes was significantly reduced from PD2 to PD42 (Fig. 4I). Collectively, these findings suggest that the departure of TEAD4 from SAAs substantially alters chromatin state transitions, thus causing transcriptional alterations in downstream SASP genes.

### TEAD4 alters the chromatin accessibility necessary for promoting senescence

**Fig. 5:**
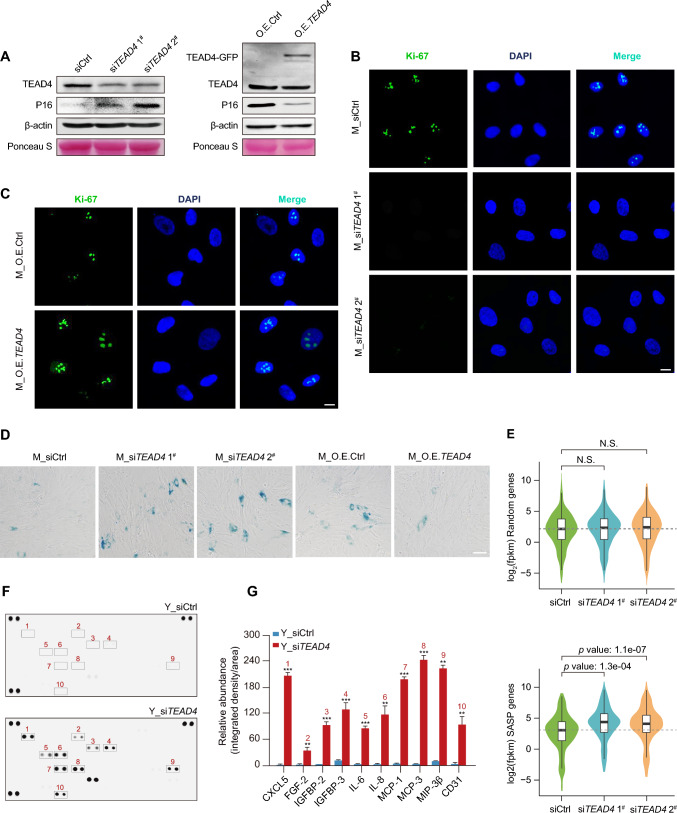
TEAD4 delays the process of senescence. **A** Immunoblotting showing the expression of P16 following knockdown or overexpression of TEAD4. PD2 cells were used for knockdown and overexpression experiments. β-actin and Ponceaus S served as loading controls. **B** and **C** Immunofluorescence staining of Ki-67 following knockdown **(B)** or overexpression **(C)** of TEAD4. The position of the nucleolus is indicated by DAPI staining. M represents middle-aged cells (PD26). Scale bar, 10 μm. **D** SA-β-gal staining of middle-aged cells following knockdown or overexpression of TEAD4. Scale bar, 50 μm. **E** Violin plots displaying the changes in expression of randomly selected genes (*n* = 350, top) and SASP genes (bottom) following TEAD4 knockdown. **F** and **G** Cytokine array analysis of secreted proteins and relative quantitation of SASP factors following TEAD4 knockdown. Error bars indicate the mean ± S.E.M of three independently performed experiments. **p < 0.01, ***p < 0.001. A Student’s *t*-test was used for statistical analysis

TEAD4 is a tumor activator with the potential to induce signaling and regulate epigenetic profiles during embryonic development and tumorigenesis [33]. As a member of the TEAD family, TEAD4 is a putative growth inhibitor involved in various cancers [34, 35]. To further uncover the role of TEAD in the modulation of senescence, we knocked down and overexpressed TEAD4 and observed the senescence phenotypes. The expression of *CDKN2A* is considered a vital biomarker of senescence. Accordingly, we found that siRNA-mediated knockdown of TEAD4 expression resulted in increased expression levels of P16, while overexpression of TEAD4 led to a decrease in P16 expression (Figs. 5A, S9A). Additionally, to investigate the role of TEAD4 during the cell cycle, we evaluated the immunofluorescence staining of Ki-67, a universal marker of proliferation. Knockdown of TEAD4 expression resulted in a decrease in Ki-67 fluorescence intensity, while overexpression of TEAD4 had the opposite effect (Figs. 5B, C, S9B). Moreover, we observed extensive and increased SA-β-gal staining after TEAD4 knockdown, which was attenuated following TEAD4 overexpression (Figs. 5D, S9C).

In particular, the genome-wide transcriptional programs uncovered a plethora of SASP genes displaying upregulated expression following TEAD4 knockdown, which was different from the unchanged expression of randomly selected genes (Figs. 5E, S9D). Validation of the transcriptional dynamics of representative SASP genes also showed that TEAD4 collapse caused a robust increase in SASP genes (Fig. S9E). In addition, GSEA analysis based on differentially expressed genes showed that SASP-related genes were upregulated following knockdown of TEAD4, while metabolic-related genes were negatively correlated with senescence, emphasizing a role for TEAD4 in the control of SASP gene expression (Fig. S9F). Alterations in SASP genes contributed to the extracellular secretion of SASP factors, thus causing dysfunction of the cellular microenvironment. Indeed, a subset of SASP factors modulated by TEAD4-induced SAAs activity was found to be accumulated following knockdown of TEAD4 expression, including IL-6, IL-8, CXCL-5, IGFBP2, IGFBP3, and MCP-3, which encode genes proven to be significantly elevated upon senescence entry (Fig. 5F, G). In conclusion, our results support the notion that TEAD4 suppresses downstream SASP gene expression and factor secretion, thus antagonizing the process of senescence.

### Disrupted chromatin accessibility following TEAD4 silencing accelerates the transcriptional program of SASP genes

**Fig. 6:**
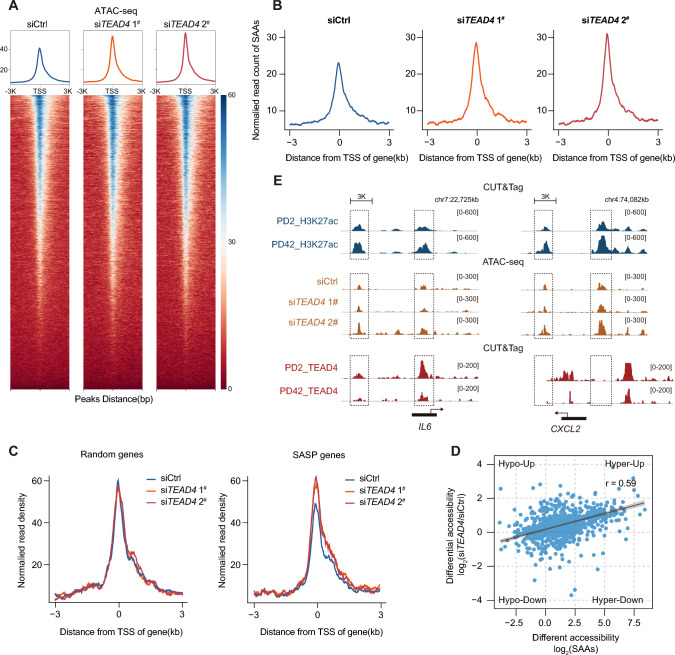
Knockdown of TEAD4 accelerates the secretion of SASP factors by modulating the flanking SAAs. **A** Heatmaps and enrichment plots showing normalized read densities of ATAC-seq signals following knockdown of TEAD4 expression. Tracks are centered at the TSS and extend ± 3 kb. **B** Enrichment of ATAC-seq peaks at SAAs following TEAD4 knockdown. Tracks are centered at the TSS and extend ± 3 kb. **C** Enrichment plots showing normalized read densities of ATAC-seq peaks at the TSS for randomly selected genes and SASP genes following knockdown of TEAD4 expression. Tracks are centered at the TSS and extend ± 3 kb. **D** Spearman’s correlation of changes in SAAs accessibility and regions that open up following TEAD4 knockdown (*r* = 0.59). **E** Integrative Genomics Viewer (IGV) snapshot showing the H3K27ac CUT&Tag and ATAC-seq, and H3K27ac RNA-seq signals at representative SASP genes, *IL6* and *CXCL2*, loci at PD2 and PD42

To determine whether the departure of TEAD4 is required to sustain an open chromatin state in SAAs, we performed ATAC-seq in stable TEAD4-knockdown cells. Overall, there were extensive changes in chromatin accessibility between the siControl and TEAD4-knockdown cells. Of note, a significant increase in accessibility was observed following knockdown of TEAD4, revealing its function in repressing global open chromatin state (Fig. 6A). Subsequently, we investigated the signal in SAAs following TEAD4 knockdown and found that silencing of TEAD4 led to a significant overall elevated chromatin accessibility in comparison with the control (Fig. 6B). Furthermore, SASP genes exhibited substantially elevated densities relative to randomly selected genes in TEAD4-knockdown cells, which is consistent with the increased SASP gene expression and factor secretion following knockdown of TEAD4 expression, further supporting the role of TEAD4 in harboring SAAs flanking SASP genes (Fig. 6C). We also assigned the SAAs with differential accessibility defined in siControl versus siTEAD4. Remarkably, the open chromatin regions in TEAD4-knockdown cells were generally correlated with accessibility changes in SAAs, which highlights the function of TEAD4 in restructuring chromatin accessibility during senescence (Fig. 6D). SAAs were always occupied with H3K27ac-decorated modifications. As an example, in comparison with young cells, *IL6* exhibited a significantly decreased enhancer signal and TEAD4 occupancy in senescent cells. Accordingly, the flanking SAA signal strength belonging to *IL6* was overactivated following TEAD4 knockdown, which is consistent with *CXCL2* (Fig. 6E). In summary, our data support a senescence system model in which a shift in TEAD4 from SAAs induces the stabilization of the open chromatin state of SAAs, resulting in a genome-wide program of downstream SASP gene expression.

## Discussion

**Fig. 7:**
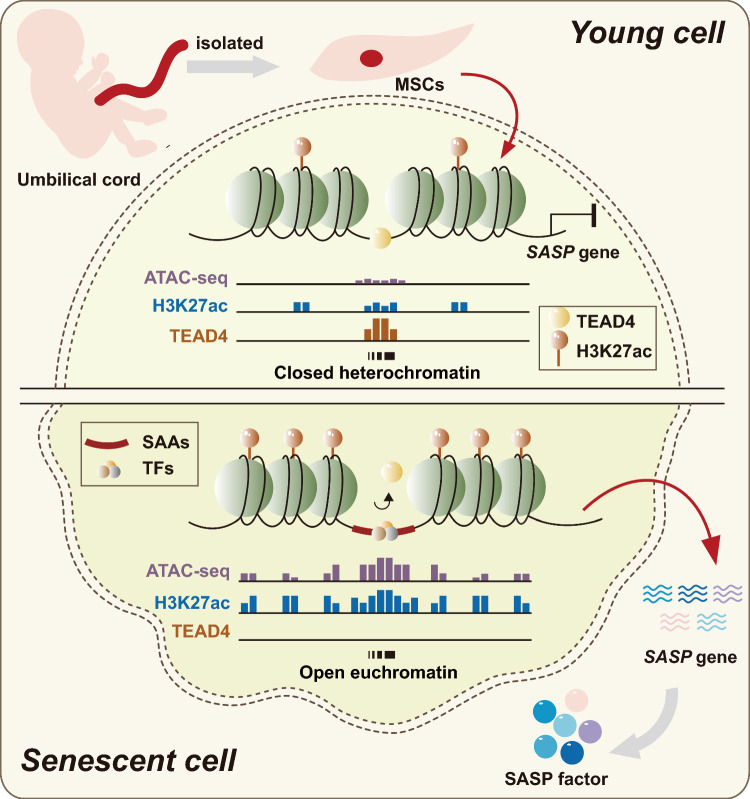
Summary schematic showing the TEAD4-mediated chromatin states, epigenetic enhancer repertoires, and SASP gene kinetics during cellular senescence. Unique senescence-activated accessibility regions (SAAs) are always resided in H3K27ac-decorated active enhancer regions. In particular, newly activated SAAs strongly trigger the activation of expression of adjacent unique SASP genes and aberrant cellular signaling relevant to SASP secretion; and importantly, the transcription factor TEAD4 is shifted away from SAAs, which enhances the strength of SAAs and induces a significant increase in SASP gene expression, ultimately leading to senescence

Senescence elicits a permanent cell cycle arrest that subsequently triggers the secretion of the bioactive secretome [36]. These profound phenotypic changes are always subject to the epigenetic pattern control of chromatin accessibility. Recent technological advances in the measurement of chromatin accessibility, such as ATAC-seq, have provided a potential association between chromatin remodeling and control of the transcriptional senescence network by allowing the quantitation of the successful enzymatic methylation of chromatin. In this report, we explored the genome-wide chromatin changes upon senescence entry by defining the redistribution of senescence-activated accessibility regions (SAAs), which always resided in H3K27ac-decorated active enhancer regions. Newly activated SAAs were tightly coupled to adjacent SASP genes; and importantly, the transcription factor TEAD4 was shifted away from SAAs, which enhanced the strength of SAAs and induced a significant increase in SASP gene expression (Fig. 7). Overall, our study uncovered a role for TEAD4 in remodeling the chromatin landscape as a potential target for downstream senescence intervention.

Even though some types of senescence show significant alterations in the chromatin landscape and share senescence effector pathways, transcription factors regulating downstream transcriptional programs in different senescence scenarios are always expressed in a tissue-specific manner. For example, studies on OIS and replicative senescence performed in distinct cells have revealed a role for the AP-1 family in reshaping open chromatin upon senescence entry, which is in accordance with the enrichment of the bZIP family (a total set of AP-1) at SAAs discovered in our study [37, 38] (Fig. 4B). Although we have subsequently excluded AP-1 function from mediating the SAA landscape, it was previously uncovered that different members, including c-JUN and ATF3, participate in chromatin remodeling, indicating the identities of the drivers involved and their possible functional combinations across types of cells and senescence. Several AP-1 factors, such as FRA1/2 and JUN, have been found to interact with TEAD4 in the co-decision of cell fate [39, 40]. Although the distinct function of these two families of transcription factors (TFs) in remodeling the chromatin landscape remains unknown, our findings support the conclusion that TFs may dynamically compete to modulate nucleosome occupancy that defines human cell fate.

Striking reports on TEAD4 have illustrated its function as an epigenetic regulator in several critical processes. TEAD4 peaks reside in active enhancers decorated with H3K27ac, while only a minor proportion of its binding sites are located in promoters, which is highly consistent with our results for TEAD4-harboring SAAs repertoires [41] (Fig. 2B, C). The recently described molecular mechanism for TF binding or departure in the accessibility landscape implies that H3K27ac can modulate DNA positioning and affect TF cooperativity by altering the interaction between the TF acidic patch and the H3 tail [42]. Hence, we examined the TEAD4 nuclear localization during senescence, and found marked departure of TEAD4 from DAPI-stained nuclei was seen in senescent cells (Fig. S10A). Although there was no significant difference in the active enhancer signal across the genome between young and senescent cells, the H3K27ac signal for TEAD4-occupied peaks was remarkably reduced in PD42 in comparison with PD2 (Fig. S10B). Meanwhile, the decreased TEAD4 peaks displayed a notable reduction in H3K27ac signal in senescent cells, while the increased TEAD4 peaks displayed relatively unchanged H3K27ac modifications between passages (Fig. S10C). It seems that the eviction of TEAD4 leads to the increased enhancer signals on SAAs regions, while the TEAD4-occupanied peaks display reduced H3K27ac decoration upon senescence entry. It might essentially owe to the uncoupling from H3K27ac modification, which supports the conclusion that TF binding and cooperativity can be regulated by histone modification which needs to be investigated further.

It has also been demonstrated that the universal enhancer-enriched property is transcriptionally active as a result of YAP/TAZ-dependent H3K27ac co-occupancy. YAP and TAZ are the most recognized coactivators of the TEAD family [43]; therefore, the dynamic recruitment of the TEAD family involved in senescence reprogramming directed us to explore the potential subsequent binding of YAP/TAZ. Although in young cells, TEAD4 and YAP interacted with each other both exogenously and endogenously, this binding was uncoupled due to the decreased expression of TEAD4 and collapse of the binding signal for TEAD4 upon senescence entry (Fig. S11A-C). Meanwhile, the senescent cells displayed relatively unchanged YAP and TAZ signal intensities during senescence in comparison with young cells using CUT&Tag, unlike the collapse of the TEAD4 signal **(**Fig. S11D). The densities of YAP and TAZ in SAAs were not significantly different between young and senescent cells, suggesting that YAP/TAZ were not involved in SAA density regulation (Fig. S11E). For further confirmation, we performed ATAC-seq analysis following stable YAP suppression and found the genome-wide chromatin accessibility peaks remained relatively unchanged after YAP knockdown, which differs from the notable increased chromatin accessibility driven by TEAD4 silencing **(**Fig. S11F, G**)**. We also examined the overlap between differentially accessible regions (DARs)—found by comparing siYAP samples with negative controls—and both SAAs and SIAs, revealing that only a fraction of these overlapped** (**Fig. S11H**)**. These data reinforce that TEAD4 uncoupling from the YAP/TAZ protein may act as a prerequisite for TEAD4-driven SAA activation, yet YAP/TAZ themselves do not participate in the activation of downstream SAAs that contributes to cellular senescence. It appears that in addition to SAAs, YAP/TAZ may collaborate with other TFs, such as the reported co-factor AP-1, to execute chromatin accessibility remodeling; however, further exploration is required.

Genome-wide analysis of chromosome accessibility and nucleosome occupancy has increased our understanding of the association between the physical and linear functional genome by uncovering a selection of putative regulatory regions throughout the genome [27]. The organization of open chromatin across promoters, enhancers, and gene bodies establishes physical interaction rules. Although promoters are often constitutively accessible, and the accessibility of distal enhancers is often restricted by cell type, our landscape of chromatin accessibility demonstrates that ATAC-seq peaks were most prevalent in enhancers, which is in accordance with a study regarding senescence in HUVECs [38]. Senescence follows a timeline that is mostly determined by the chromatin landscape. The steady relationship between gene expression and chromatin accessibility during senescence reveals a potential regulatory mechanism in which chromatin rearrangement patterns control specific gene expression during senescence. Our research, conducted utilizing a variety of high-throughput sequencing techniques, sheds light on the dynamic interaction among chromatin accessibility, enhancer repertoires, and gene expression. Understanding the chromatin-reshaping role of the senescence suppressor TEAD4 is made possible by characterizing genome-while open chromatin dynamics. Uncovering the mechanisms of SASP component regulation is necessary to understand the manner by which the physiology and behavior of senescence are regulated and, eventually, to develop therapeutic strategies.

### Electronic supplementary material

Below is the link to the electronic supplementary material.

Supplementary file1 (PDF 4832 kb)

## Data Availability

All related sequencing data have been uploaded to the NCBI Gene Expression Omnibus and are accessible through the accession number GSE226326.
