# Supplementary material for: TEAD4 antagonizes cellular senescence by remodeling chromatin accessibility at enhancer regions
Source: Cell Mol Life Sci. 2023 Oct 19;80(11):330. doi: 10.1007/s00018-023-04980-9 (PMC10587282; doi:10.1007/s00018-023-04980-9)
Supplement: Supplementary file 1 — Supplementary file1 (PDF 4832 kb) [file 18_2023_4980_MOESM1_ESM.pdf]

# TEAD4 antagonizes cellular senescence by remodeling chromatin accessibility at enhancer regions

Donghui Zhang[1†], Yanmei Zhu[1†], Yanmin Ju[2†], Hongyong Zhang[1], Xiaopeng Zou[1], Shangrong She[2], Danping Zhu[2] and Yiting Guan[1*]

**SUPPORTING INFORMATION**

SUPPLEMENTARY TABLE ........................................................................ 2

Primers used in H3K27ac ChIP-qPCR ........................................................ 2

Primers used in TEAD4 ChIP-qPCR ........................................................... 3

Primers used in RT-qPCR ............................................................................ 4

Primers used in luciferase assay ................................................................ 5

siRNA sequence used in knock down expriment ....................................... 5

Antibodies .................................................................................................... 5

SUPPLEMENTARY FIGURES AND LEGENDS ........................................... 6

**SUPPLEMENTARY TABLE**

## Primers used in H3K27ac ChIP-qPCR

| | | |
|---|---|---|
| IGF2-R-1 | For: 5’-CACCGCGTCAACATACCAGG-3’ | Positive control Cai Y, et al., Nat Commun. 2021 |
| | Rev: 5’-CATGTGTGATTCGTGCCTTGC-3’ | |
| *IGF2*-R-2 | For: 5’-AAGCAAGGAAGTCACGGGTC-3’ | |
| | Rev: 5’-GAGAAATAGGGCTTCGGGCG-3’ | |
| *CDKN1A*-e-1 | For: 5’-GACTGGTGCTGGCTGAAAGG-3’ | |
| | Rev: 5’-TTCCAAGGCTGGGTCATAAA-3’ | |
| *CDKN1A*-e-2 | For: 5’-TTGTTGCCTGTTGTTCCAAT-3’ | |
| | Rev: 5’-GAGCACCAATAATGCCTCTT-3’ | |
| *CDKN1A*-e-3 | For: 5’-TTGTTGCCTGTTGTTCCAAT-3’ | |
| | Rev: 5’-GAGCACCAATAATGCCTCTT-3’ | |
| *CDKN1A*-e-4 | For: 5’-ACGAGCCCTCAGTCTTCTTG-3’ | |
| | Rev: 5’-GGAGATACCACCACTGTCAAA-3’ | |
| *CDKN1A*-e-5 | For: 5’-AAGATGGCTATGTCGGTGAA-3’ | |
| | Rev: 5’-CTGGGTCTGATATGAAACTCG-3’ | |
| IGF2-R-3 | For: 5’-GGGCATCTCTGTCATGGTGG-3’ | Positive control Cai Y, et al., Nat Commun. 2021 |
| | Rev: 5’-GGCATTTGGGATACACCCGT-3’ | |
| IGF2-R-4 | For: 5’-GCGAGGTAAACCTCCCAGAG-3’ | |
| | Rev: 5’-CGGGTCTGGTGATGCCATAG-3’ | |
| *EMP1*-e-1 | For: 5’-GAAAGGAAAGGGAAGGAATA-3’ | |
| | Rev: 5’-CAAGAAGTCTACTAAGGCAGGT-3’ | |
| *EMP1*-e-2 | For: 5’-CTTTCTATGGCACCACTCTG-3’ | |
| | Rev: 5’-GTTTCCACGCCTACTACCTG-3’ | |
| *EMP1*-e-3 | For: 5’-GAAGGTATAACATGGTCGTG-3’ | |
| | Rev: 5’-TTGGGAATCATAATAGCAAC-3’ | |
| *EMP1*-e-4 | For: 5’-GCAGGTCCCAGTGAGTGTTG-3’ | |
| | Rev: 5’-TCATGGATGGAGCTGAAAGC-3’ | |
| *EMP1*-e-5 | For: 5’-GGCTGGGAGTAGGAGATGGG-3’ | |
| | Rev: 5’-GGAGGCAGAAGTGGGAGGAT-3’ | |

**Primers used in TEAD4 ChIP-qPCR**

| | | |
|---|---|---|
| *CTGF* | For: 5’-CTGGCAATGTCCTGACAAAA-3’ | Positive control, Guo Y, et al., Nat Commun. 2022 |
| | Rev: 5’-CTGGCTGATCTCAAGTGCTG-3’ | |
| *CXCL1*-e | For: 5’-CATTCGTAACTATTAGGGACT-3’ | |
| | Rev: 5’-GTGAAAGAGGAGGAACAAGG-3’ | |
| *CXCL2*-e | For: 5’-GAATGAATCCTGGAGCTGTT-3’ | |
| | Rev: 5’-GTTTAGTCTTGGGAGGGTGT-3’ | |
| *CXCL5*-e | For: 5’-GGTCTGGCAGTCCAATGAAG-3’ | |
| | Rev: 5’-GGGCTCACTGATTTGCTCCT-3’ | |
| *IL6*-e | For: 5’-TATTCCACATTGGTGCTTAC-3’ | |
| | Rev: 5’-GTCTGGTTGTATCTCCCTCA-3’ | |
| *IGFBP1*-e | For: 5’-CTGGATGAAGGGACTGTGGT-3’ | |
| | Rev: 5’-ATTCTTCTTGGATGTGGGAG-3’ | |
| *IGFBP2*-e | For: 5’-CAGGCTGCTCTTGAACTCAT-3’ | |
| | Rev: 5’-GTCCACCCACTCTGCTTACT-3’ | |
| *CXCL8*-e | For: 5’-TGCCACTGTATCCTAACCTG-3’ | |
| | Rev: 5’-ATTTGTCTCACCCACTTGAC-3’ | |
| *MMP9*-e | For: 5’-AAAGTCAGGCATCAGTATTCAA-3’ | |
| | Rev: 5’-GGTATAACAAGTGTCTCGTGGG-3’ | |
| *CCL2*-e | For: 5’-GACTATCTGGGTGGGTAGGG-3’ | |
| | Rev: 5’-AGAGGACTGGCGTAGAGGTT-3’ | |
| *CCL3*-e | For: 5’-TAGGTACAGAATGATAGGGCAATT-3’ | |
| | Rev: 5’-TCTTGAATCTGGGAGGTGGA-3’ | |
| *CCL7*-e | For: 5’-CCACCAACAGTAAACAAAGA-3’ | |
| | Rev: 5’-GTATCACCTCACCTCCATTA-3’ | |
| *SPP1*-e | For: 5’-TTGAGATGGGAGACAAGAAA-3’ | |
| | Rev: 5’-TAGCATAGCACCGATGAAGC-3’ | |

**Primers used in RT-qPCR**

| | |
|---|---|
| *CDKN2A* | For: 5’-GATCCAGGTGGGTAGAAGGTC-3’ |
| | Rev: 5’-CCCCTGCAAACTTCGTCCT-3’ |
| *CDKN1A* | For: 5’-TGTCCGTCAGAACCCATGC-3’ |
| | Rev: 5’-AAAGTCGAAGTTCCATCGCTC-3’ |
| *EMP1* | For: 5’-GTGCTGGCTGTGCATTCTTG-3’ |
| | Rev: 5’-CCGTGGTGATACTGCGTTCC-3’ |
| *TEAD1* | For: 5’-ATGGAAAGGATGAGTGACTCTGC-3’ |
| | Rev: 5’-TCCCACATGGTGGATAGATAGC-3’ |
| *TEAD2* | For: 5’-CTTCGTGGAACCGCCAGAT-3’ |
| | Rev: 5’-GGAGGCCACCCTTTTTCTCA-3’ |
| *TEAD3* | For: 5’-TGGACCCTCTCAGGACATCAA-3’ |
| | Rev: 5’-CCAGGGGCTCATAACTGCTG-3’ |
| *TEAD4* | For: 5’-GAACGGGGACCCTCCAATG-3’ |
| | Rev: 5’-GCGAGCATACTCTGTCTCAAC-3’ |
| *CXCL1* | For: 5’-TGCTGCTCCTGCTCCTGGTA-3’ |
| | Rev: 5’-TGTGGCTATGACTTCGGTTTGG-3’ |
| *CXCL5* | For: 5’-AGCTGCGTTGCGTTTGTTTAC-3’ |
| | Rev: 5’-AGCTGCGTTGCGTTTGTTTAC-3’ |
| *IL6* | For: 5’-ACTCACCTCTTCAGAACGAATTG-3’ |
| | Rev: 5’-CCATCTTTGGAAGGTTCAGGTTG-3’ |
| *IGFBP1* | For: 5’-TTTTACCTGCCAAACTGCAACA-3’ |
| | Rev: 5’-CCCATTCCAAGGGTAGACGC-3’ |
| *IGFBP2* | For: 5’-TGCACATCCCCAACTGTGAC-3’ |
| | Rev: 5’-TGTAGAAGAGATGACACTCGGG-3’ |
| *CXCL8* | For: 5’-ACTGAGAGTGATTGAGAGTGGAC-3’ |
| | Rev: 5’-AACCCTCTGCACCCAGTTTTC-3’ |
| *MMP9* | For: 5’-AGACCTGGGCAGATTCCAAAC-3’ |
| | Rev: 5’-CGGCAAGTCTTCCGAGTAGT-3’ |
| *CCL2* | For: 5’-CAGCCAGATGCAATCAATGCC -3’ |
| | Rev: 5’-TGGAATCCTGAACCCACTTCT-3’ |
| *CCL7* | For: 5’-CAGCTGCTTTCAGCCCCCAGGGGCT-3’ |
| | Rev: 5’-TGGCTACTGGTGGTCCTTCT-3’ |
| *SPP1* | For: 5’-GAAGTTTCGCAGACCTGACAT-3’ |
| | Rev: 5’-GTATGCACCATTCAACTCCTCG-3’ |
| *GAPDH* | For: 5’-GACTAACCCTGCGCTCCTG-3’ |
| | Rev: 5’-GCCCAATACGACCAAATCAG-3’ |

**Primers used in luciferase assay**

| | |
|---|---|
| E1 | For: 5'- GGTACCTTAGGAGAAGTGACGTTCTGTCATT-3' |
| | Rev: 5'- CTCGAGAATGACAGAACGTCACTTCTCCTAA-3' |
| E2 | For: 5'- GGTACCGTTCCTGTCATCAGTGGGTA-3' |
| | Rev: 5'- CTCGAGTATTCTTGGCATAGCATCTT-3' |
| E3 | For: 5'-GGTACCAAATCAAGCCAAGAAGGGCAACA-3' |
| | Rev: 5'-CTCGAGGAGGGAAGGCACCCATCAGC-3' |
| E4 | For: 5'- GGTACCAAGTGGGCTGAAGCAGGTGA-3' |
| | Rev: 5'- CTCGAGCCTGTGAGCGGCTGTTGTAG-3' |
| E5 | For: 5'- GGTACCCCCACCCTCACCCTCCAACA-3' |
| | Rev: 5'- AAGCTTGGAAGGCAGCAGGCAACACC-3' |

**siRNA sequence used in knock down expriment**

| | |
|---|---|
| si*TEAD4* #1 | 5′-GGAACAAACUGUGCCUGAATT-3′ |
| si*TEAD4* #2 | 5′-GCUUGUGGAUGAAGUUGATTT-3′ |
| si*YAP* #2 | 5′-GUGGGACUCAAAAUCCAGUTT-3′ |

**Antibodies**

| | | |
|---|---|---|
| TEAD4 | Proteintech | 12418-1-AP |
| IgG | Proteintech | 30000-0-AP |
| P16 | Proteintech | 10883-1-AP |
| IgG H&L (HRP) | Abcam | ab205718 |
| H3K27ac | Abcam | ab4729 |
| Ki-67 | Abcam | ab15580 |
| β-actin | Abcam | ab8226 |
| IRDye 800CW anti-mouse | LI-COR | 926-32210 |
| IRDye 800CW anti-rabbit | LI-COR | 92632211 |
| YAP | Cell Signaling Technology | 14074 |
| TAZ (WWTR1) | Sigma Aldrich | HPA007415 |

# SUPPLEMENTARY FIGURES AND LEGENDS

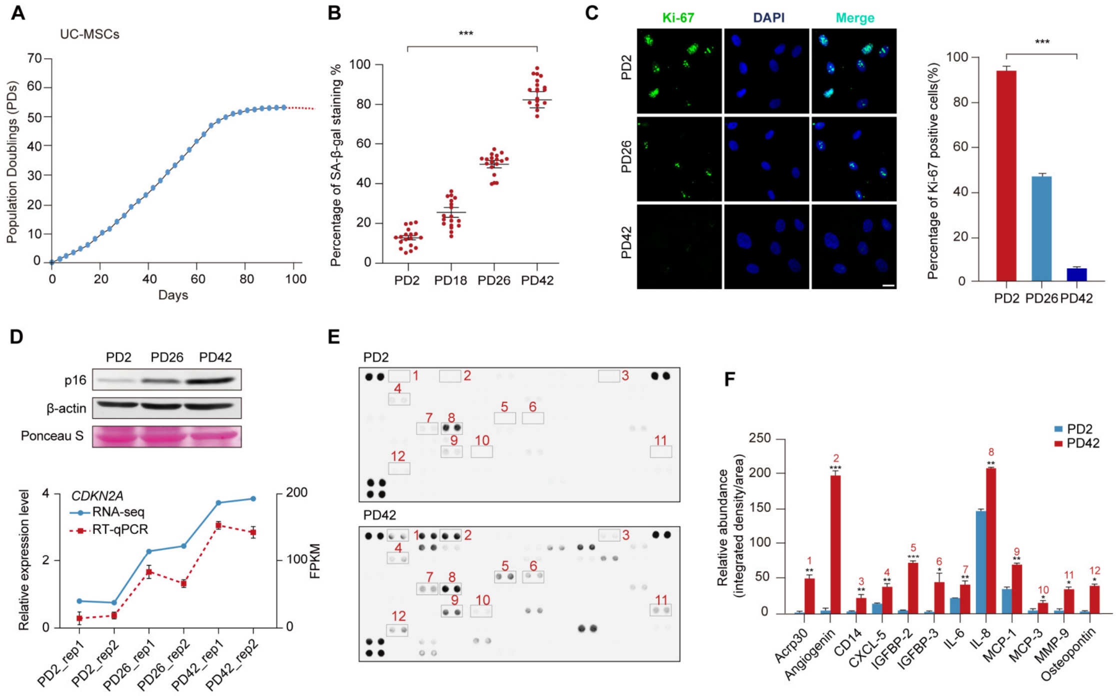

**Fig. S1** Creation of an *in vitro* replicative senescence model. **A** Population doubling of primary human umbilical cord mesenchymal stem cells (UC-MSCs). **B** Percentage of SA-β-gal staining in young to senescent UC-MSCs. Error bars indicate the mean ± S.E.M. of three independently performed experiments. ****p* < 0.001. A Student's *t*-test was used for statistical analysis. **C** Immunofluorescence staining and statistics of Ki-67 in UC-MSCs. The nucleolus is indicated by DAPI staining. Scale bar, 10 µm. Error bars indicate the mean ± S.E.M. of three independently performed experiments. ****p* < 0.001. **D** Top: Immunoblotting of P16 expression in young to senescent UC-MSCs. β-actin and ponceaus S were used as the loading controls. Bottom: *CDKN2A* expression in PD2, PD26, and PD42 UC-MSCs. The dashed line indicates RT-qPCR, while the solid line indicates RNA-seq data. Error bars indicate the mean ± S.E.M. of three independently performed experiments. **E** and **F** Cytokine array analysis of secreted proteins and relative quantitation of SASP factors during senescence. Error bars indicate the mean ± S.E.M of three independently performed experiments. **p* < 0.05, ***p* < 0.01, ****p* < 0.001. A Student's *t*-test was used for statistical analysis.

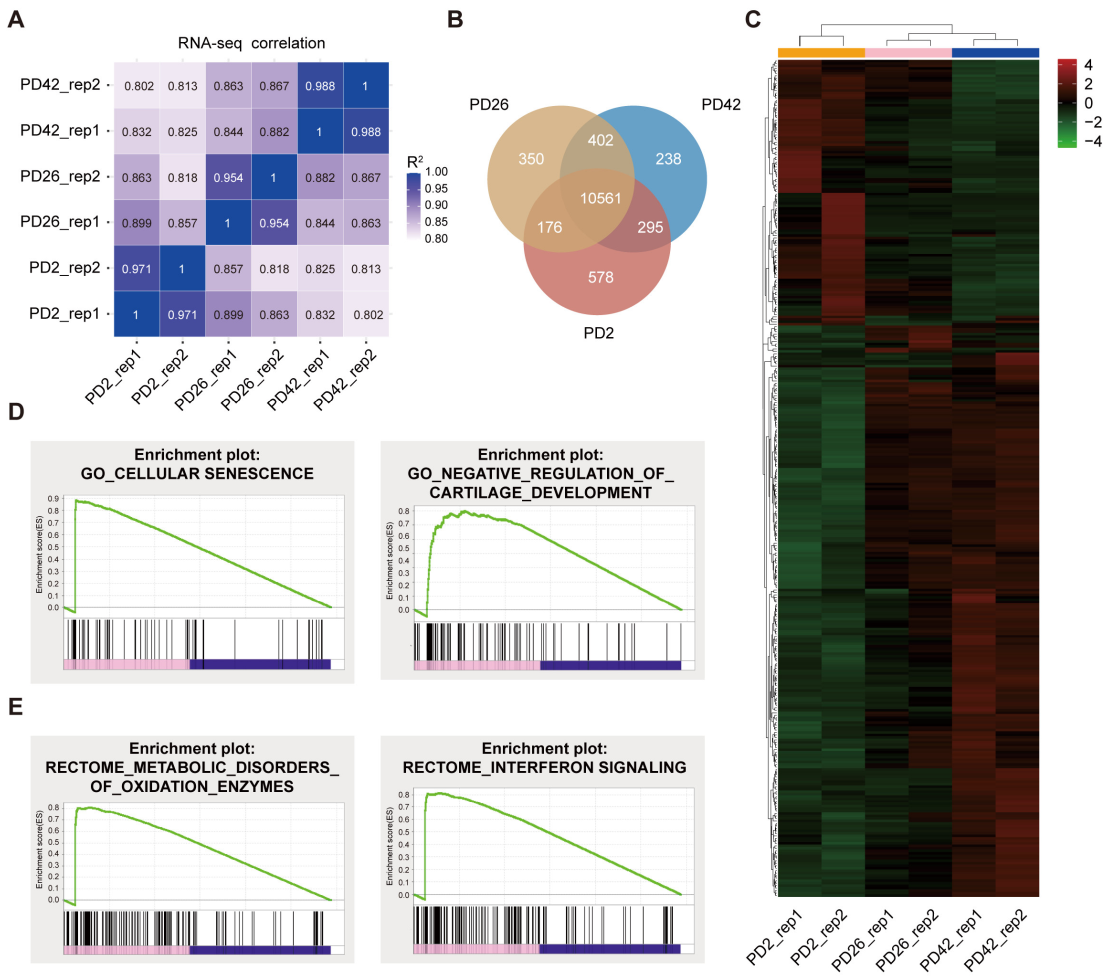


**Fig. S2** Exploration of genome-wide transcriptional programs upon senescence entry. **A** Correlation heatmap of RNA-seq data. There are two independently performed biological replicates for each passage number (rep 1 and 2). **B** Venn diagram indicating the number of differentially expressed genes in in young to senescent UC-MSCs. **C** Heatmap of RNA-seq data showing hierarchically clustered gene expression in young to senescent UC-MSCs. **D** and **E** GSEA showing the enrichment of GO **(D)** and RECTOME **(E)** pathways in PD2 versus PD42 cells.

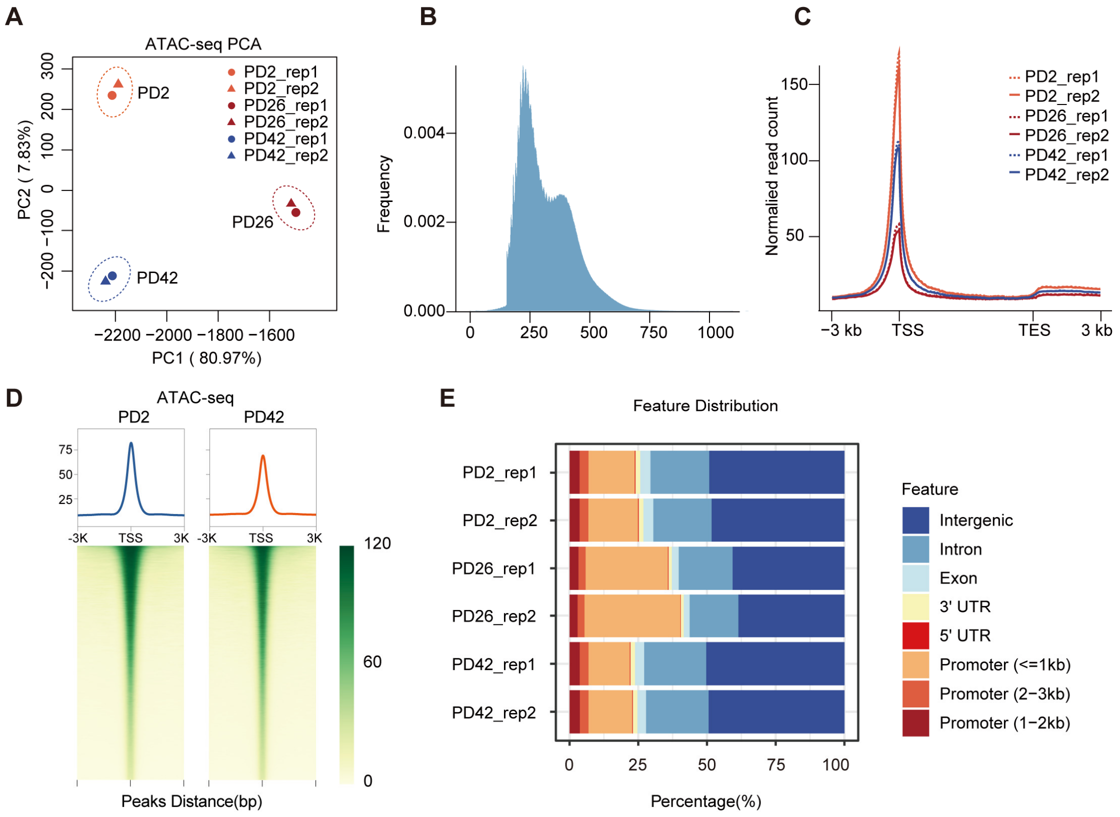


**Fig. S3** Remodeling of the chromatin landscape occurs during senescence. **A** Principal Component Analysis (PCA) displaying the correlation and dispersion of ATAC-seq in young to senescent UC-MSCs. There were two independently performed biological replicates for each passage number (rep 1 and 2). **B** Representative distribution of insert size, showing clear signal modulation for mono- and di-nucleosomes. **C** Normalized ATAC-seq read count across the genome. Following peak calling, the mean ATAC-seq peaks present between the TSS (−3 kb) and the TES (+3 kb) were calculated across the genome. TSS, transcription start site; TES, transcription end site. **D** Heatmap and enrichment plots showing normalized read densities for ATAC-seq peaks at PD2 and PD42. Tracks are centered at the peaks and extend ± 3 kb. **E** Genomic feature distributions of accessible chromatin regions that changed during senescence. UTR, untranslated region.

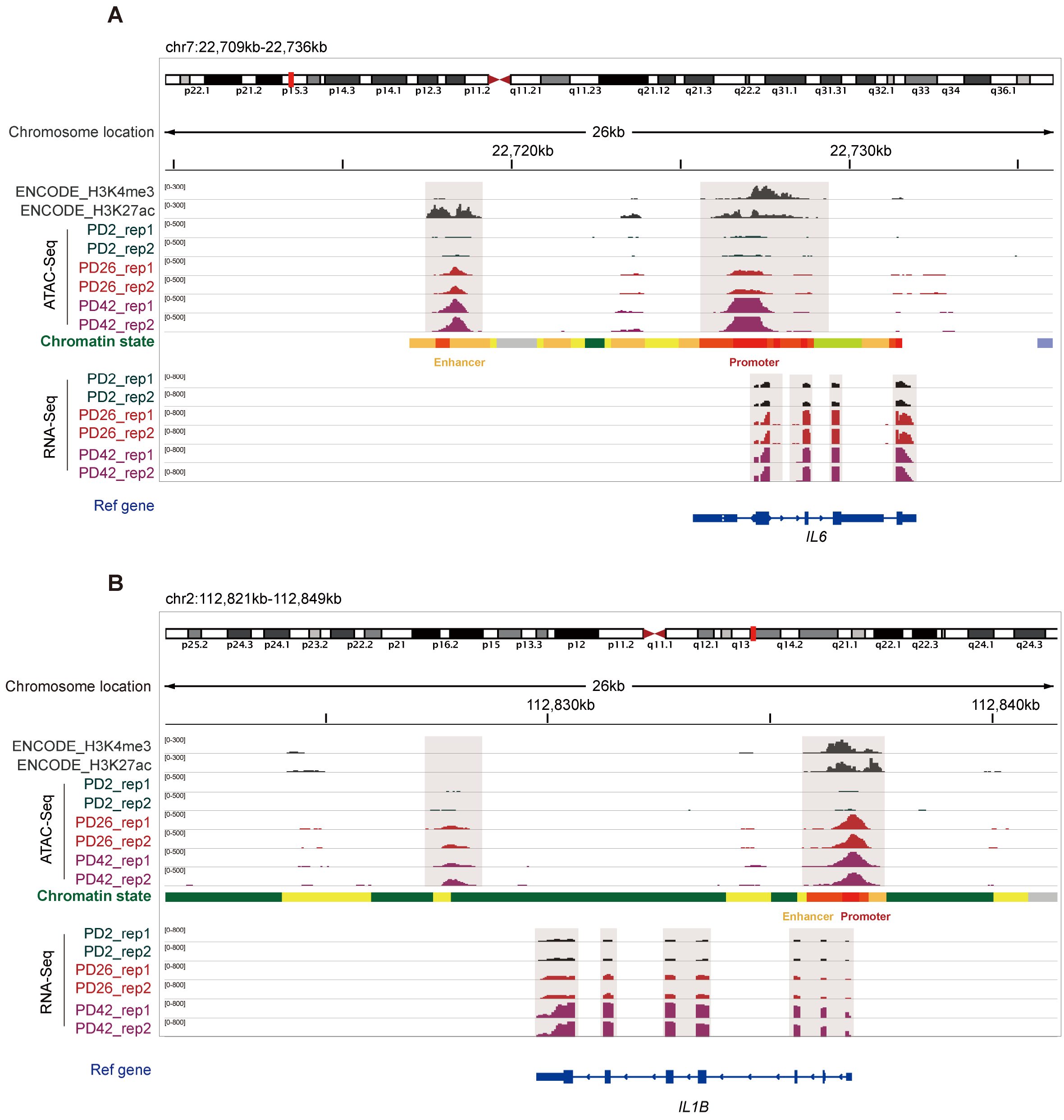


**Fig. S4** ATAC-seq data are highly similar to public Encyclopedia of DNA Elements (ENCODE) data for enhancers decorated by histone modification H3K27ac. **A** and **B** Integrative Genomics Viewer (IGV) snapshot displaying the H3K4me3 and H3K27ac peaks of MSCs from ENCODE, in addition to our ATAC-seq and RNA-seq at the *IL6* and *IL8* loci in young to senescent UC-MSCs. Vertical gray boxes indicate enhancer and promoter ATAC-seq peaks. Chromatin states were obtained from ENCODE: yellow, weak enhancer; orange, strong enhancer; red, active promoter; green, transcribed region; and gray, heterochromatin.

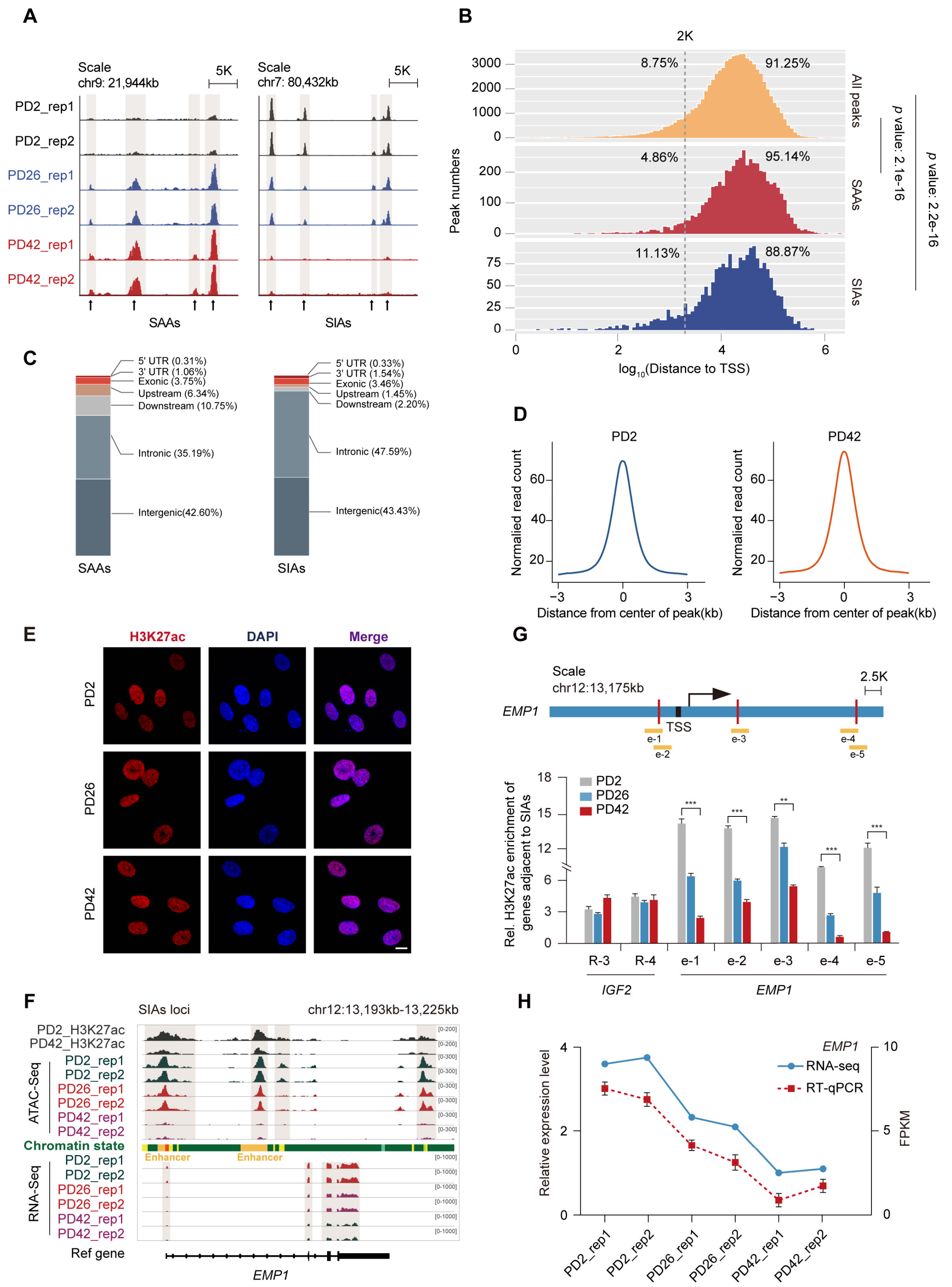


**Fig. S5** Enrichment analysis of differentially accessible regions during senescence. **A** Insertion tracks of senescence-activated accessibility regions (SAAs) and senescence-inactivated accessibility regions (SIAs) at loci on chromosomes 7 and 9. **B** Distribution of the distances between SAA or SIA ATAC-seq peaks and the TSS. **C** Annotations of SAAs and SIAs showing genomic features of the differentially accessible regions. **D**

Enrichment of H3K27ac-driven CUT&Tag peaks at PD2 and PD42. Tracks are centered at the peaks and extend ± 3 kb. **E** Immunofluorescence staining for H3K27ac in UC-MSCs. The nucleolus is indicated by DAPI staining. Scale bar, 10 μm. **F** Snapshot displaying the H3K27ac, ATAC-Seq, and RNA-seq profiles in young to senescent UC-MSCs at the representative SIA region of the *EMP1* locus. Vertical gray boxes indicate ATAC-seq peaks for enhancer and promoter regions and the corresponding *EMP1* expression. **G** Location diagram of H3K27ac ChIP-qPCR primers within the *EMP1* locus (top). ChIP-qPCR data showing the relative H3K27ac levels in SIAs within *EMP1* in young to senescent UC-MSCs (bottom). H3K27ac enrichment in *IGF2* was used as the positive control and IgG was used as the negative control. Enrichment was normalized to a 1:10 dilution of input. Error bars indicate the mean ± S.E.M. of three independently performed experiments. One-way ANOVA followed by Dunnett's multiple comparisons test was used for statistical analysis. N.S., not significant, *$p < 0.05$, ***$p < 0.001$. **H** The expression of *EMP1* in young to senescent UC-MSCs. The dashed line indicates RT-qPCR, while the solid line indicates RNA-seq data. Error bars indicate the mean ± S.E.M. of three independently performed experiments.

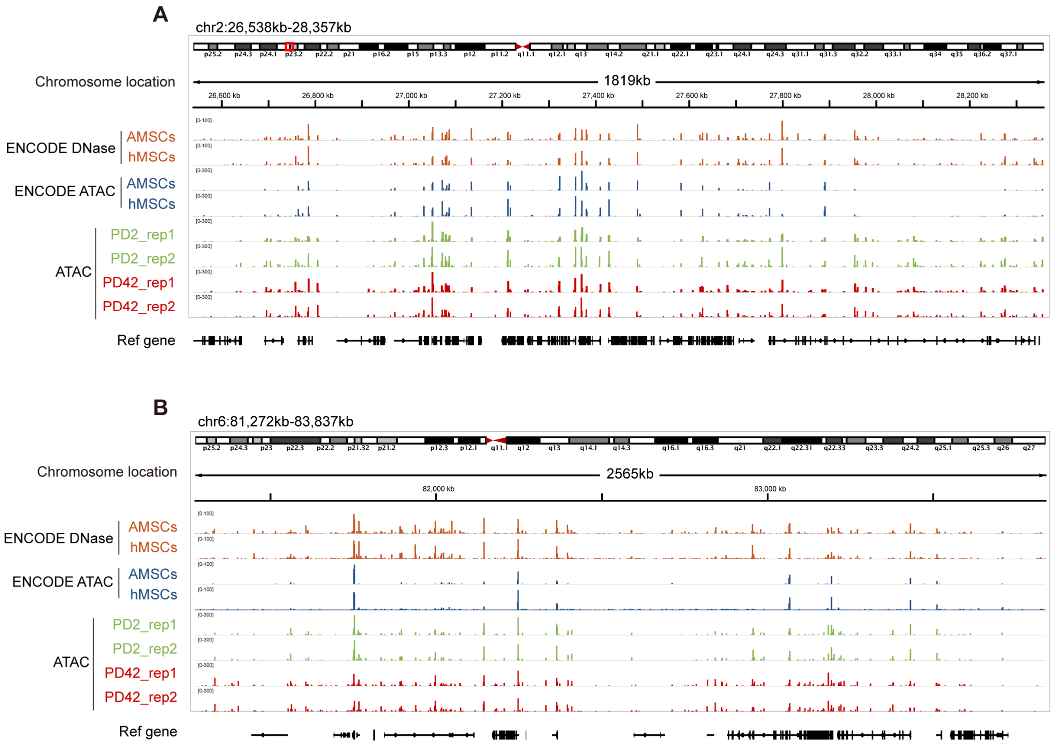

**Fig. S6** Comparison between DNase-seq and ATAC-seq data from ENCODE and other types of mesenchymal stem cells and our chromatin accessibility data. **A** and **B** Integrative Genomics Viewer (IGV) snapshot displaying the DNase-seq and ATAC-seq peaks of AMSCs and hMSCs from ENCODE and the ATAC-seq peaks in young to senescent UC-MSCs at loci on chromosomes 2 and 6.

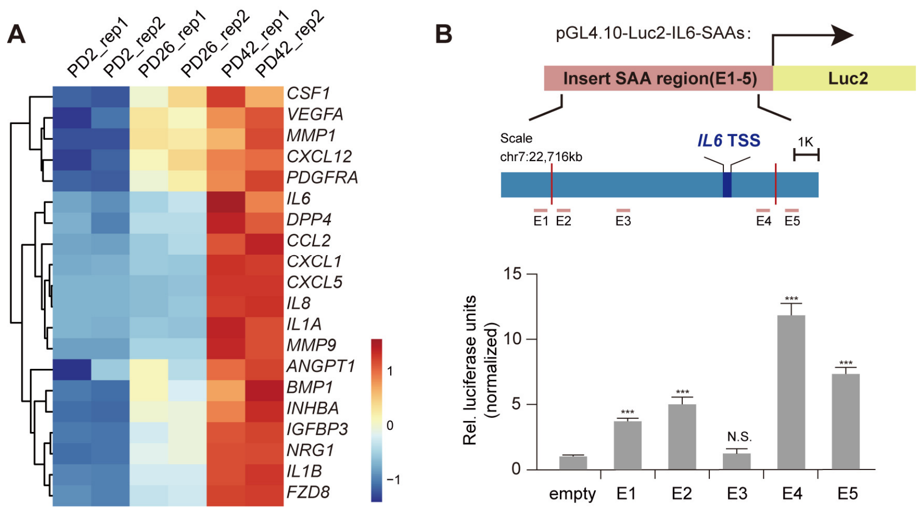


**Fig. S7** SAAs regulate the expression of adjacent SASP genes. **A** Heatmaps displaying normalized gene expression among PD2, PD26, and PD42. **B** Location diagram of the SAA primers (E1-E5) within *IL6* locus (top); the activity of E1-E5 was measured using the dual-luciferase reporter assay in HEK 293T cells (bottom). Error bars indicate the mean ± S.E.M. of three independently performed experiments. A Student's *t*-test was used for statistical analysis. N.S., not significant, ***$p < 0.001$.

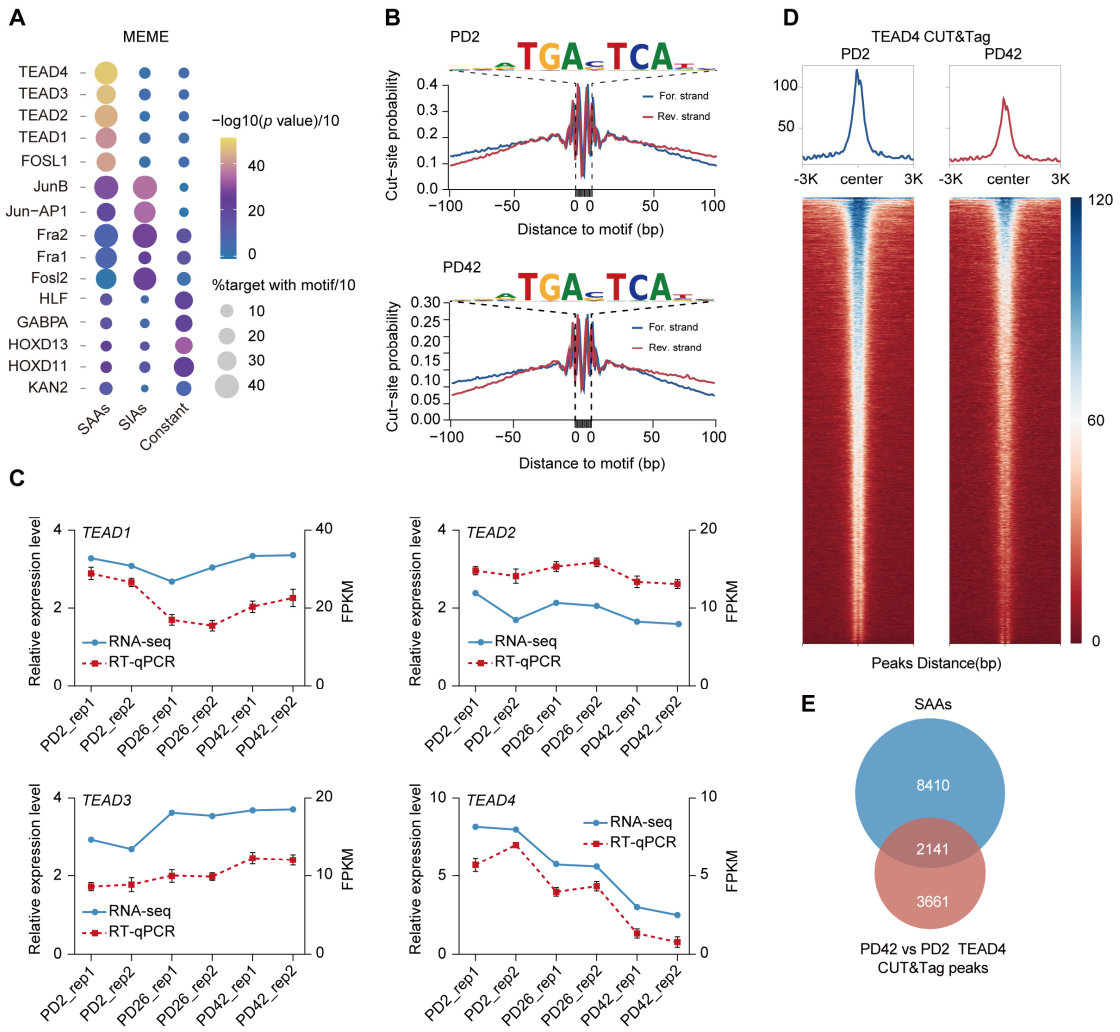


**Fig. S8** TEAD4 binding at SAAs accelerates the transcription of SASP genes. **A** Top-ranked enriched motifs from MEME motif analysis of SAAs and SIAs. The circle size represents the percentage of regions with the motif and the color represents the *p* value (shrink to 10×). **B** ATAC-seq pattern in the bZIP motif at PD2 and PD42. Normalization was performed to ensure that each motif possessed the same mean number of insertions 200–500 bp away. **C** Expression of other TEAD family members, *TEAD1*, *TEAD2*, and *TEAD3* in young to senescent UC-MSCs. The dashed line indicates RT-qPCR, while the solid line indicates RNA-seq data. Error bars indicate the mean ± S.E.M. of three independently performed experiments. **D** Heatmaps and enrichment plots showing normalized read densities for TEAD4 CUT&Tag peaks performed at PD2 and PD42. Tracks are centered at the peaks and extend ± 3 kb. **E** Venn diagram showing overlap of TEAD4 CUT&Tag peaks between PD42 and PD2, in addition to SAA signals defined in ATAC-seq peaks.

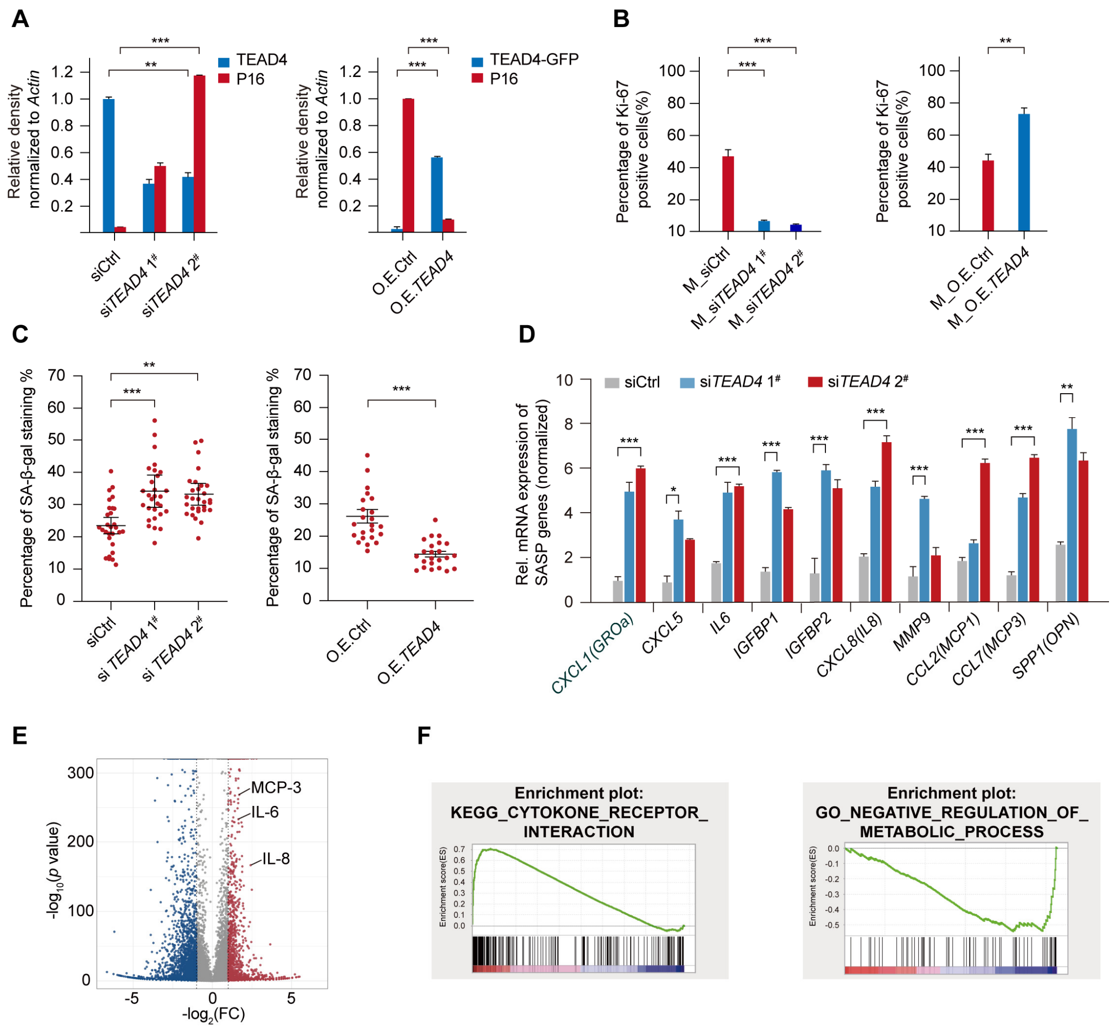


**Fig. S9** Suppression of TEAD4 leads to the upregulation of SAA-adjacent SASP genes. **A** Relative density of immunoblotting showing the expression of P16 following knockdown or overexpression of TEAD4. β-actin served as controls. Error bars indicate the mean ± S.E.M. of three independently performed experiments. $^{**}p < 0.01$, $^{***}p < 0.001$. **B** Immunofluorescence statistics of the expression of Ki-67 following knockdown or overexpression of TEAD4. Error bars indicate the mean ± S.E.M. of three independently performed experiments. $^{**}p < 0.01$, $^{***}p < 0.001$. **C** Percentage of SA-β-gal staining following TEAD4 suppression or overexpression. Error bars indicate the mean ± S.E.M. of three independently performed experiments. $^{**}p < 0.01$, $^{***}p < 0.001$. A Student's *t*-test was used for statistical analysis. **D** RNA-Seq scatter diagram showing the gene expression profiles of control versus siTEAD4 cells. Representative SASP genes are labeled. **E** RT-qPCR data showing the relative expression of SASP genes following downregulation of TEAD4 expression. Error bars indicate the mean ± S.E.M. of three independently performed experiments. $^{*}p < 0.05$, $^{**}p < 0.01$, $^{***}p < 0.001$. One-way ANOVA followed by Dunnett's multiple

comparison test was used for statistical analysis. **F** GSEA showing the enrichment of SASP-related pathways in control versus siTEAD4 cells.

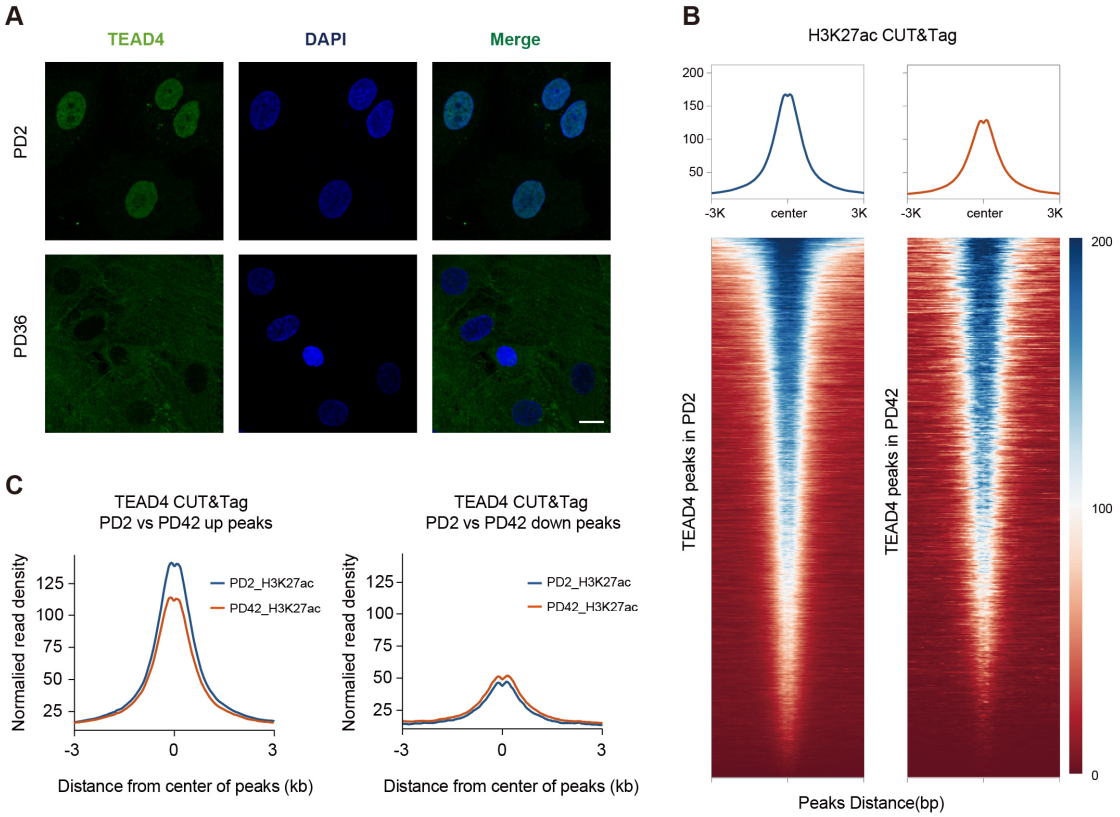


**Fig. S10** The departure of TEAD4 from H3K27ac modification is necessary for establishments of SAAs. **A** Immunofluorescence staining of TEAD4 in young and middle-senescent cells. The nucleolus is indicated by DAPI staining. Scale bar, 10 μm. **B** Heatmap and enrichment plots showing normalized read densities for H3k27ac CUT&Tag of TEAD4-occupanied peaks at PD2 and PD42. Tracks are centered at the peaks and extend ± 3 kb. **C** Enrichment plots showing normalized read densities for H3k27ac CUT&Tag peaks of differentially TEAD4-occupanied peaks at PD2 and PD42. Tracks are centered at the peaks and extend ± 3 kb.

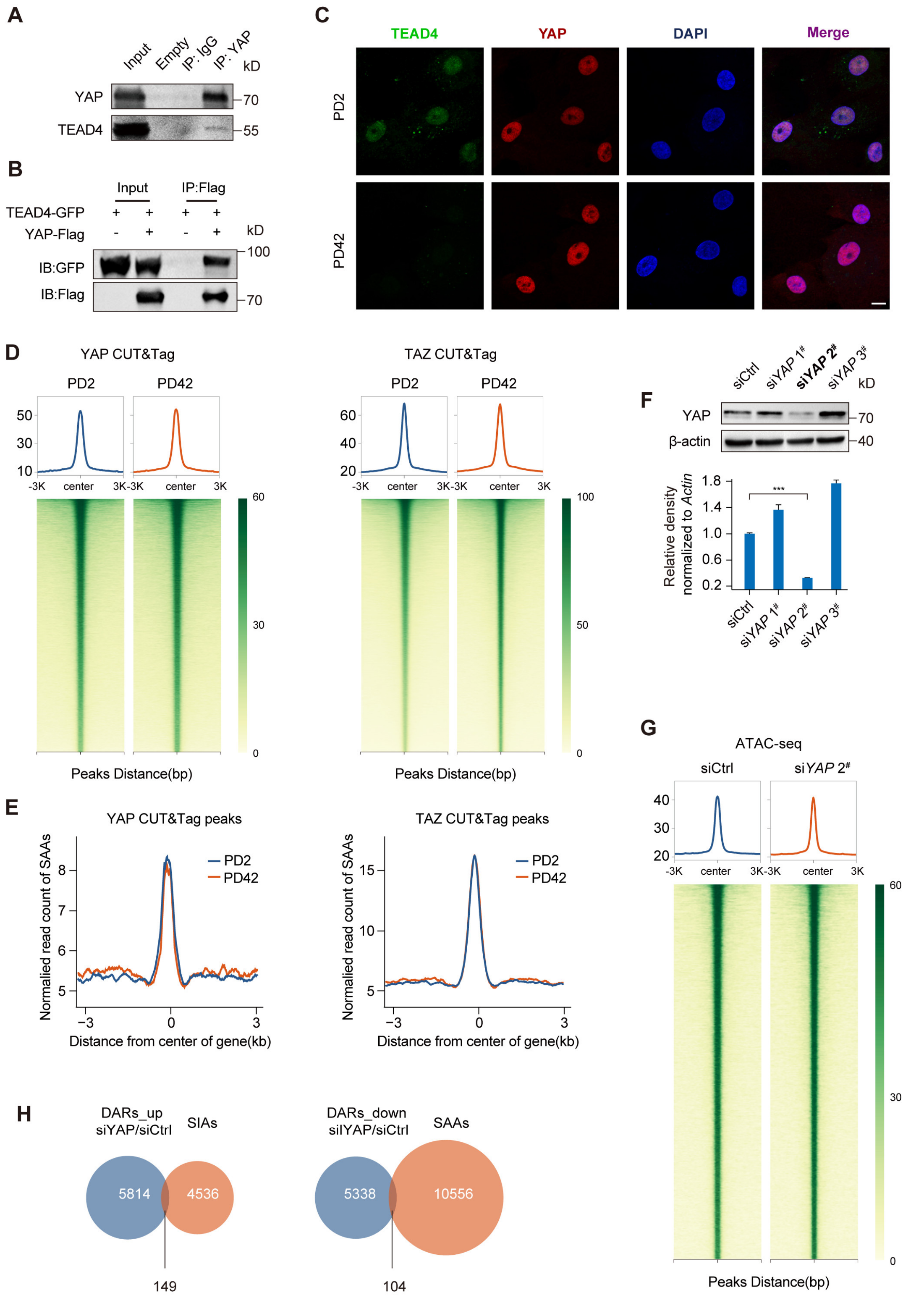


**Fig. S11** YAP/TAZ are not involved in the regulation of TEAD4-mediated SAAs flanking SASP genes. **A** Lysates from PD2 were subjected to immunoprecipitation (IP) with an anti-YAP antibody and subsequent immunoblotting with the indicated antibodies. **B** Lysates of HEK293T cells co-overexpressing pcDNA3.1-YAP-Flag and pSIN-TEAD4-GFP were subjected to IP and subsequent immunoblotting with anti-Flag

and anti-GFP antibodies. **D** Heatmaps and enrichment plots showing normalized read densities of YAP and TAZ CUT&Tag signals in PD2 and PD42. Tracks are centered at the peaks and extend ± 3 kb. **E** Enrichment of YAP and TAZ CUT&Tag signals in PD2 and PD42. Tracks are centered at the peaks and extend ± 3 kb. **F** Immunoblotting analysis and quantitation showing the expression of YAP following knockdown of TEAD4 by using small-interfering RNA (siRNAs) in PD10. β-actin served as the loading control. ***$p$ < 0.001. **G** Heatmaps and enrichment of ATAC-seq peaks following YAP knockdown. Tracks are centered at the peaks and extend ± 3 kb. **H** Venn diagram showing the overlap of differentially expressed chromatin accessible region (DAR)-defined ATAC-seq peaks following YAP knockdown, with the SAAs and SIAs peaks defined in PD2 and PD42.
